# The E3 ubiquitin ligase MGRN1 targets melanocortin receptors MC1R and MC4R via interactions with transmembrane adapters

**DOI:** 10.1242/jcs.264084

**Published:** 2025-12-09

**Authors:** Pragya Parashara, Lei Gao, Alyssa Riglos, Dorothy Lartey, Sonia B. Sidhu, Tessa Marks, Carys Williams, Grace Siauw, Kai-Jing Lee, Anna I. L. Ostrem, Christian Siebold, Michael Riffle, Maia Kinnebrew, Teresa M. Gunn, Jennifer H. Kong

**Affiliations:** ^1^ Department of Biochemistry, School of Medicine, University of Washington, Seattle, WA 98195, USA; ^2^ Division of Structural Biology, Wellcome Centre for Human Genetics, Nuffield Department of Medicine, University of Oxford, Oxford, OX3 7BN, UK; ^3^ Department of Genome Sciences, School of Medicine, University of Washington, Seattle, WA 98195, USA; ^4^ Department of Biochemistry, Stanford University School of Medicine, Stanford, CA 94305, USA; ^5^ McLaughlin Research Institute and Touro University College of Osteopathic Medicine, Great Falls, MT 59405, USA

**Keywords:** MGRN1, E3 ubiquitin ligase, Transmembrane adapter proteins, Melanocortin receptor, Receptor regulation, GPCR trafficking

## Abstract

Mahogunin ring finger 1 (MGRN1) is a membrane-tethered E3 ligase that fine-tunes signaling sensitivity by targeting surface receptors for ubiquitylation and degradation. Although MGRN1 is known to regulate the Hedgehog signaling effector Smoothened (SMO) via the transmembrane adapter multiple epidermal growth factor-like 8 (MEGF8), the broader scope of its regulatory network has been speculative. Here, we identify attractin (ATRN) and attractin-like 1 (ATRNL1) as additional transmembrane adapters that recruit MGRN1 and regulate cell surface receptor turnover. Through co-immunoprecipitation, we show that ATRN interacts with the RING domain of MGRN1. Functional assays suggest that ATRN and ATRNL1 work with MGRN1 to promote the ubiquitylation and degradation of the melanocortin receptors MC1R and MC4R, in a process analogous to its regulation of SMO. Loss of MGRN1 or ATRN leads to increased surface and ciliary localization of MC4R in fibroblasts and elevated MC1R levels in melanocytes, resulting in enhanced eumelanin production. These findings expand the known repertoire of MGRN1-regulated receptors and provide new insight into a shared mechanism by which membrane-tethered E3 ligases utilize transmembrane adapters to facilitate substrate receptor specificity.

## Teaser

**Summary:** The E3 ligase MGRN1 uses transmembrane adapters ATRN and ATRNL1 to ubiquitylate and degrade melanocortin receptors, regulating receptor turnover and signaling sensitivity.

## INTRODUCTION

Mahogunin ring finger 1 (MGRN1) is a multifunctional E3 ubiquitin ligase involved in diverse cellular processes, including mitochondrial quality control, endosomal trafficking and the regulation of signaling pathways. In mouse models, loss-of-function mutations in *Mgrn1* lead to severe phenotypes, including left–right patterning defects, congenital heart malformations, embryonic lethality, craniofacial abnormalities, pigmentation defects, late-onset spongiform neurodegeneration, mitochondrial dysfunction and partial suppression of diet-induced obesity (Bagher et al., 2006; Cota et al., 2006; Groza et al., 2023; Gunn et al., 2013, 2019; He et al., 2003; Jiao et al., 2009b; Kong et al., 2020; Miller et al., 1997; Mukherjee and Chakrabarti, 2016b; Overton and Leibel, 2011; Phan et al., 2002; Sun et al., 2007). Despite these findings, little is known about how this versatile E3 ligase identifies and targets specific substrates for ubiquitylation. One area of particular interest is how MGRN1 regulates surface receptor abundance. Although the precise mechanism remains incompletely understood, we know that MGRN1 and its vertebrate-specific paralog RNF157 represent a novel pair of membrane-tethered E3 ligases that interact with surface-bound transmembrane adapters to selectively ubiquitylate receptors within the plasma membrane. We previously demonstrated that MGRN1 binds to the cytoplasmic tail of the single-pass transmembrane adapter multiple epidermal growth factor-like 8 (MEGF8) to promote the degradation of the G-protein coupled receptor (GPCR) Smoothened (SMO) within the Hedgehog signaling pathway (Kong et al., 2020; Pusapati et al., 2018; Williams et al., 2025 preprint). However, the wide range of phenotypes observed in *Mgrn1*-deficient mice extends beyond defects in Hedgehog signaling, suggesting that MGRN1 might also regulate other surface receptors mediated through interactions with additional transmembrane adapters.

The melanocortin receptor (MCR) family consists of five GPCRs: MC1R, MC2R, MC3R, MC4R and MC5R, which are expressed in different tissues. These receptors regulate a broad spectrum of physiological processes, including pigmentation, the stress response, energy homeostasis and appetite control. Epistasis experiments in mice have shown that MGRN1 inhibits MC1R and MC4R signaling (Miller et al., 1997; Overton and Leibel, 2011; Phan et al., 2002), with prior studies reporting interactions between MGRN1 and MC1R, MC2R and MC4R (Cooray et al., 2011; Pérez-Oliva et al., 2009). However, conflicting data have left the mechanisms underlying this inhibition unclear. Studies in mice suggest that MGRN1 regulates MC1R through its E3 ubiquitin ligase activity (Gunn et al., 2013). In contrast, other studies propose that MGRN1 modulates MCR function independently of ubiquitylation, possibly by competing with Gαs proteins for receptor binding (Pérez-Oliva et al., 2009). These discrepancies underscore the need to clarify how MGRN1 modulates MCR signaling.

To address this gap, we provide evidence that MGRN1 interacts with transmembrane adapters to modulate the surface abundance of MC1R and MC4R via ubiquitylation, utilizing a mechanism similar to its regulation of SMO (Kong et al., 2020). Using mass spectrometry and biochemical assays, we demonstrate that MGRN1 interacts with the transmembrane adapters attractin (ATRN) and attractin-like 1 (ATRNL1). Through ubiquitylation assays, we show that ATRN and ATRNL1 facilitate MGRN1-mediated ubiquitylation and degradation of MC1R and MC4R. Furthermore, we show that the loss of *Mgrn1* or *Atrn* results in elevated surface and ciliary MC4R levels in NIH/3T3 fibroblast cells. Similarly, in melanocytes, a loss of *Mgrn1* or *Atrn* leads to increased surface MC1R and enhanced signaling activity, as measured by elevated eumelanin production. Collectively, these findings support a new model in which MGRN1 functions as a membrane-tethered E3 ligase, interacting with ATRN and ATRNL1 to regulate surface GPCR abundance and fine-tune signaling sensitivity in receiving cells.

## RESULTS

### MGRN1 interacts with ATRN and ATRNL1

**Fig. 1.**
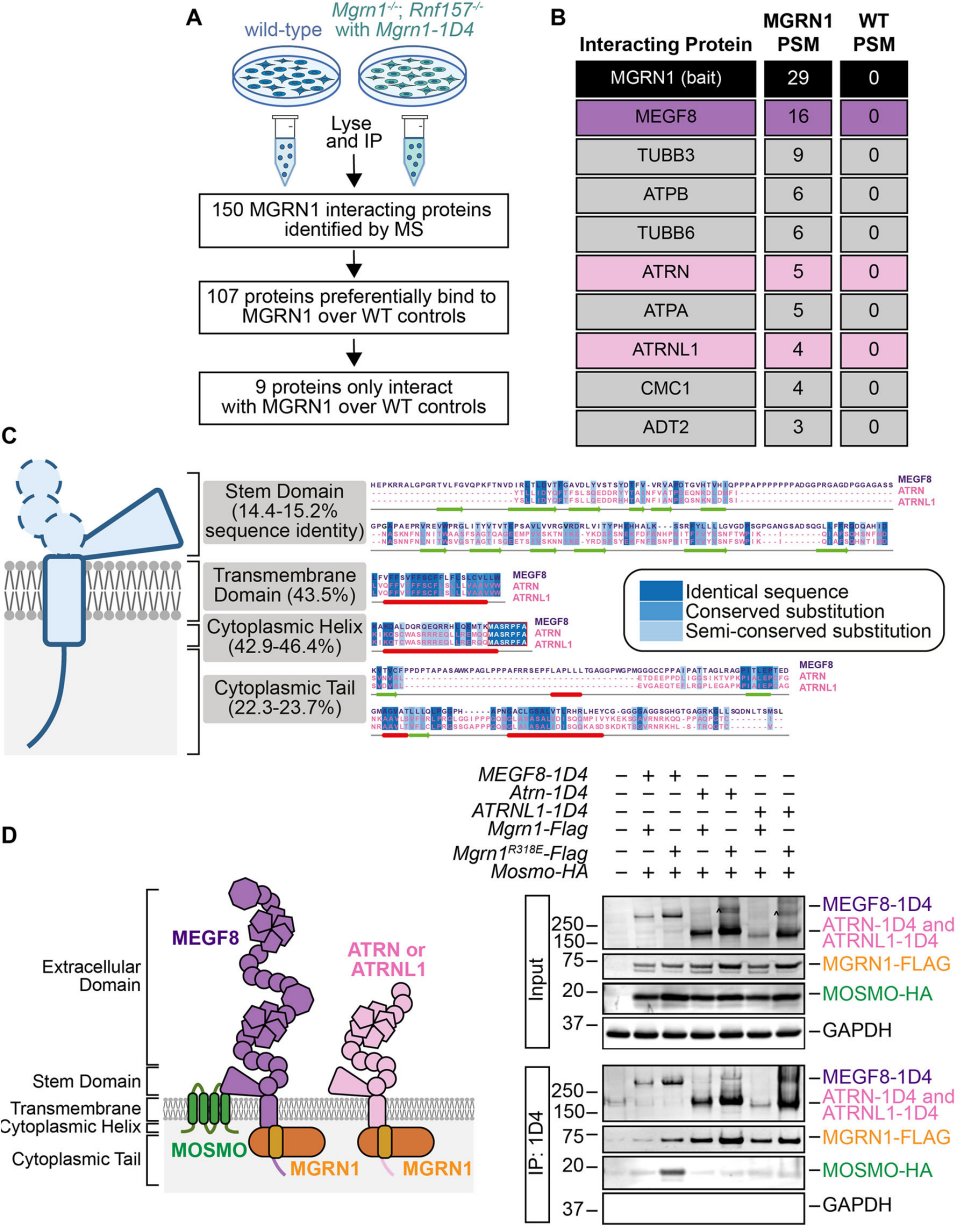
**Identification of MGRN1-interacting proteins.** (A) Schematic of the co-immunoprecipitation mass spectrometry (co-IP/MS) strategy used to identify MGRN1-interacting proteins. (B) Table listing nine proteins that interact exclusively with MGRN1 compared to wild-type controls. PSM, Peptide spectrum match. (C) Multiple sequence alignment (MSA) of the stem domain, transmembrane domain, cytoplasmic helix and cytoplasmic tail of MEGF8, ATRN and ATRNL1. MSA was performed using ClustalO and visualized with Jalview. Sequence identities between MEGF8, ATRN and ATRNL1 were calculated using ClustalO. Secondary structure prediction of MEGF8 was generated with JPred, where helices are represented by thick red underlining and sheets by green arrows. The highly conserved MASRPFA motif observed within the cytoplasmic helix of MEGF8, ATRN and ATRNL1 is boxed in red. (D, left) Schematic representation of the MOSMO–MEGF8–MGRN1 (MMM) and the ATRN–MGRN1 or ATRNL1–MGRN1 complexes. (D, right) Co-IP analysis of MEGF8, ATRN and ATRNL1 interactions. HEK293T cells were transiently transfected with 1D4-tagged *MEGF8*, *Atrn* or *ATRNL1*, and FLAG-tagged *Mgrn1* (wild-type or catalytically compromised *Mgrn1^R318E^*) and HA-tagged *Mosmo*. Proteins were immunoprecipitated using anti-1D4 beads and analyzed by western blotting. MGRN1 interacts with MEGF8, ATRN and ATRNL1, whereas MOSMO interacts specifically with only MEGF8. The carets (^) indicate a likely ATRN and ATRNL1 dimer or oligomer, as observed in previous studies (Walker, 2010). GAPDH serves as a loading control. Blot representative of three independent repeats.

MGRN1 and its vertebrate-specific paralog RNF157 are among a few known E3 ubiquitin ligases that interact with membrane-bound transmembrane adapters to modulate the abundance of surface-bound signaling receptors (Lebensohn et al., 2022). MGRN1 and RNF157 exhibit functional redundancy, with RNF157 being able to compensate for the loss of MGRN1 in certain cell types (Kong et al., 2020). Thus, to identify additional MGRN1 adapters, we performed co-immunoprecipitation experiments followed by mass spectrometry (co-IP/MS) using *Mgrn1^−/−^*; *Rnf157^−/−^* NIH/3T3 cells stably expressing MGRN1 fused to a 1D4 tag (MGRN1–1D4) (Fig. 1A). This approach revealed nine proteins that interacted exclusively with MGRN1 compared to wild-type controls (Fig. 1B). These interacting proteins included tubulin subunits (TUBB3 and TUBB6), ATP synthase subunits (ATPA and ATPB, also known as ATP5F1A and ATP5F1B, respectively), and mitochondrial proteins (CMC1 and ADT2; also known as SLC25A12 and SLC25A5, respectively), supporting the known roles of MGRN1 in regulating microtubule dynamics (Mukherjee et al., 2017; Srivastava and Chakrabarti, 2014) and maintaining mitochondrial homeostasis (Gunn et al., 2019; Mukherjee and Chakrabarti, 2016a,b; Sun et al., 2007). Importantly, our analysis identified multiple epidermal growth factor-like 8 (MEGF8), which we previously demonstrated forms a complex with MGRN1 (Kong et al., 2020; Williams et al., 2025 preprint), and two additional surface proteins, ATRN and ATRNL1 (Fig. 1B).

We analyzed the sequence similarity between MEGF8, ATRN, and ATRNL1 to understand the basis of their interaction with MGRN1. In humans and mice, ATRN and ATRNL1 are paralogs of MEGF8 (Nagle et al., 1999). Sequence alignment revealed that most of their shared regions were within the transmembrane and cytoplasmic domains, with a 43.5% shared sequence identity in the transmembrane domain and 42.9–46.4% in the cytoplasmic helix (Fig. 1C). Notably, the highly conserved cytoplasmic motif (MASRPFA), which we speculate provides the conformational flexibility required for bound MGRN1 to ubiquitylate receptor substrates (Williams et al., 2025 preprint), is also adjacent to the cytoplasmic helix of ATRN and ATRNL1 (Nagle et al., 1999; Walker et al., 2007) (Fig. 1C). Previous studies have provided evidence supporting an interaction between ATRN and MGRN1. Fluorescently labeled ATRN and MGRN1 colocalize in HEK293T cells and interactions have been observed by co-immunoprecipitation (co-IP) experiments conducted in Neuro2A neuroblastoma cells (Walker, 2010). Additionally, a recent micropublication has shown that the MASRPF motif is required for the interaction between *Drosophila* MGRN1 and Distracted, the fly ortholog of ATRN and ATRNL1, reinforcing the importance of this motif (Nawaratne et al., 2021).

Based on these reports and our co-IP/MS data, we hypothesized that MGRN1 interacts with ATRN and ATRNL1 through their intracellular domains (Nawaratne et al., 2021). To test this, we performed interaction assays in HEK293T cells expressing tagged versions of these proteins. Co-IP experiments confirmed that MGRN1 interacts with MEGF8, ATRN and ATRNL1 (Fig. 1D). To increase the expression levels of MGRN1, MEGF8, ATRN and ATRNL1 for co-IP, we included a catalytically compromised *Mgrn1^R318E^* construct, which fails to ubiquitylate itself and its binding partners. The R318E substitution disrupts the allosteric ‘linchpin’ residue within the RING domain, which is required to engage and stabilize the E2-ubiquitin conjugate, thereby promoting efficient ubiquitin transfer (Lips et al., 2020; Pruneda et al., 2012). This mutation markedly reduces E3 ligase activity while preserving the overall RING structure (Williams et al., 2025 preprint). Consistent with this reduction in catalytic activity, stable addback of *Mgrn1^R318E^* in *Mgrn1^−/−^;Rnf157^−/−^* NIH/3T3 cells results in sustained Hedgehog pathway activation in the absence of Hedgehog ligands or agonists, as measured through elevated ciliary SMO (Fig. S1A,B) and increased *Gli1* expression (Fig. S1C). Interestingly, our data revealed that ATRN and ATRNL1 did not interact with MOSMO, despite their similarity to MEGF8. This lack of interaction is likely due to a substantial divergence in the extracellular stem domains of MEGF8 and ATRN or ATRNL1, a region that interacts extensively with MOSMO in recently published structure and is crucial for MOSMO binding (Kong et al., 2021; Williams et al., 2025 preprint). Collectively, these findings expand upon the known membrane-bound adapters of MGRN1 and simultaneously highlight the unique interaction profiles of MEGF8, ATRN and ATRNL1.

### ATRN interacts with the RING domain of MGRN1

*Mgrn1*- and *Atrn*-null mutant mice share strikingly similar phenotypes, including a dark coat color due to the loss of a subapical band of pheomelanin on individual hairs and an elevated basal metabolic rate that confers resistance to diet-induced obesity (Blanchard et al., 1995; Dinulescu et al., 1998; Gunn et al., 2001; He et al., 2001; Miller et al., 1997; Nagle et al., 1999; Overton and Leibel, 2011; Phan et al., 2006). In addition, extensive epistasis experiments in mice have demonstrated that both MGRN1 and ATRN function as negative regulators of melanocortin signaling (He et al., 2001; Miller et al., 1997; Phan et al., 2002). However, the precise mechanism by which MGRN1 and ATRN modulate melanocortin signaling remains unresolved.

**Fig. 2.**
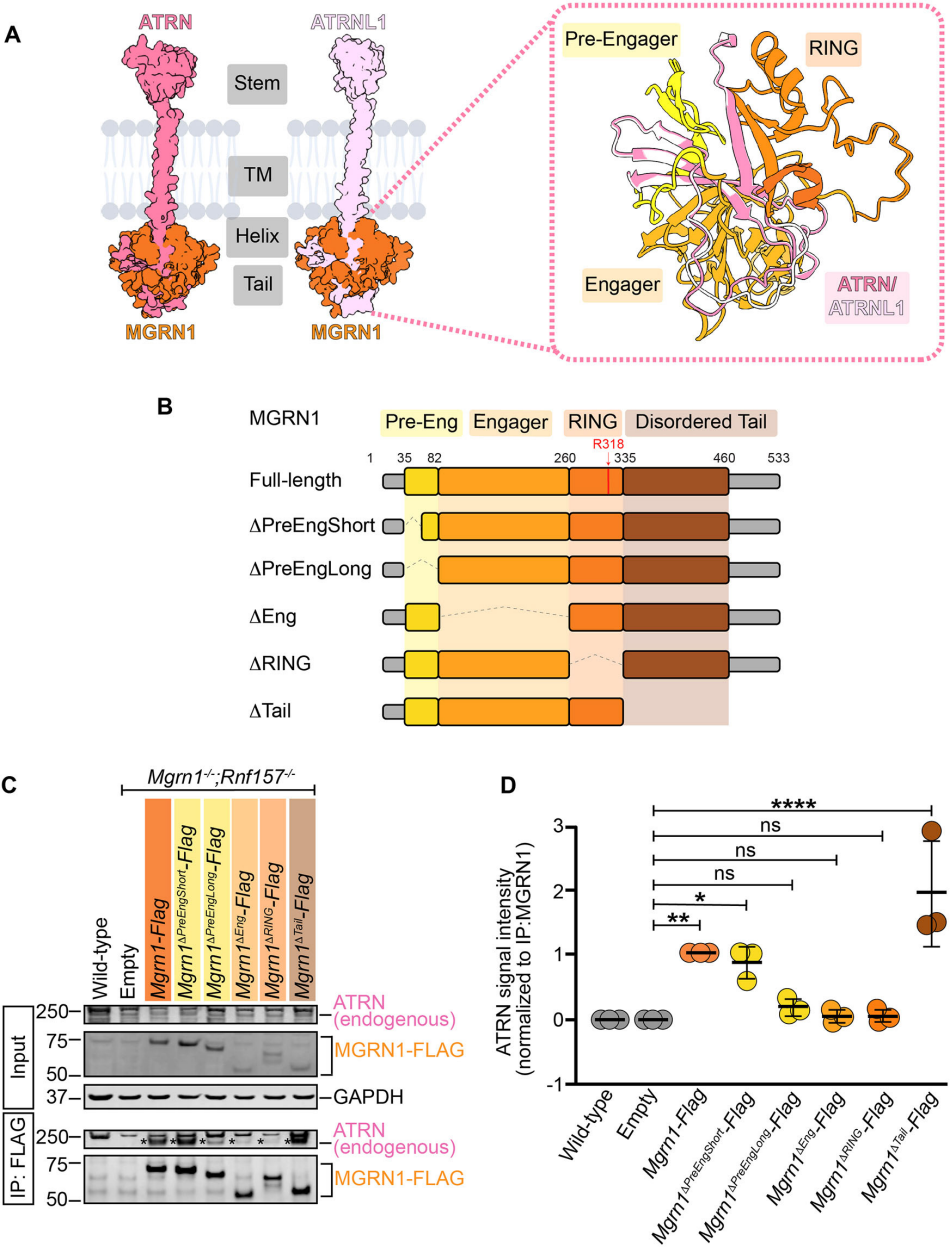
**ATRN interacts with the RING domain of MGRN1.** (A, top left) AlphaFold-predicted ATRN–MGRN1 and ATRNL1–MGRN1 interactions. Protein structural models were computed using the AlphaFold2 Google Colab (Mirdita et al., 2022) using ATRN (UniProt ID: O75882), ATRNL1 (UniProt ID: Q5VV63) and MGRN1 (UniProt ID: O60291) as inputs. Both ATRN (residues 1157–1429, dark pink) and ATRNL1 (residues 1107–1379, light pink) contain four distinct domains, which we refer to as the extracellular stem (Stem), transmembrane (TM), cytoplasmic helix (Helix) and cytoplasmic tail (Tail). In interaction models, MGRN1 (orange) interacts with the cytoplasmic helix and tail of ATRN and ATRNL1. (A, top right) AlphaFold prediction of ATRN and ATRNL1 interacting with the MGRN1 pre-engager (yellow), engager (light orange) and RING (dark orange) domains. (B) Schematic of the domain organization of MGRN1 (533 aa) and the large deletions generated for the interaction assays. MGRN1 has four previously described interaction and functional domains, which we refer to as the pre-engager (PreEng), engager (Eng), RING and intrinsically disordered tail (Tail). The red arrow indicates the location of the R318E point mutation within the RING domain. (C) Co-IP analysis of the ATRN–MGRN1 interactions in wild-type and *Mgrn1^−/−^*; *Rnf157^−/−^* NIH/3T3 cells stably expressing FLAG-tagged wild-type *Mgrn1* or one of several *Mgrn1* truncation mutants. The mutants include deletions of the Pre-engager (*ΔPreEngShort* and *ΔPreEngLong,* residues 35–48 and 35–82, respectively), Engager (*ΔEng*, residues 79–260), RING (*ΔRING*, residues 260–335) and disordered tail (*ΔTail*, residues 335-460). Proteins were immunoprecipitated using anti-FLAG beads and analyzed by western blotting. The expression of MGRN1 constructs in addback cell lines was verified using an anti-FLAG antibody. GAPDH serves as a loading control. The asterisks (*) indicate the endogenous monomeric band of ATRN observed in the Flag IPs that was used for quantification. (D) Quantification of endogenous ATRN western blot signal intensities relative to wild-type and truncated MGRN1 in immunoprecipitated samples. The graph represents data from three independent experiments, with each dot representing one experimental replicate and with horizontal lines denoting the mean±s.d. Statistical significance was determined using one-way ANOVA with multiple comparisons. *P<0.05; **P<0.01; ****P<0.0001; ns, not significant.

To determine which domain of MGRN1 is responsible for interacting with ATRN, we generated a series of FLAG-tagged MGRN1 truncation mutants, targeting previously described interaction and functional domains (Blanchard et al., 1995; Chakrabarti and Hegde, 2009; Chhangani and Mishra, 2013; Guerra et al., 2013; Jiao et al., 2009a; Kuramoto et al., 2001; Pérez-Oliva et al., 2009; Phan et al., 2006). These constructs included the deletion of regions immediately preceding the engager domain (ΔPreEngShort and ΔPreEngLong), the engager domain (ΔEng), the RING domain (ΔRING) and the intrinsically disordered tail (ΔTail) (Fig. 2A,B). Using a Flp-In system, we stably expressed either a wild-type or truncated version of *Mgrn1* in *Mgrn1^−/−^*; *Rnf157^−/−^* NIH/3T3 cells. Co-IP with anti-FLAG beads followed by probing for endogenous ATRN revealed that wild-type MGRN1 associated robustly with endogenous ATRN (Fig. 2C,D). By contrast, ATRN binding was markedly reduced with the MGRN1^ΔPreEngLong^, MGRN1^ΔEng^ and MGRN1^ΔRING^ mutants (Fig. 2C,D). The importance of these three domains is consistent with the AlphaFold prediction of the ATRN–MGRN1 interface (Fig. 2A) and closely parallels the multipartite contacts revealed by the cryo-electron microscopy structure of MGRN1 bound to the intracellular domain of MEGF8 (Williams et al., 2025 preprint). Collectively, these findings highlight a conserved mode of engagement that underlies how this membrane-tethered E3 ligase interacts with multiple transmembrane adapters. Notably, although MGRN1 has been reported to interact with several proteins, MEGF8 and ATRN are the first identified partners that engage with the RING domain. This was an unexpected observation, given that RING domains are classically known for catalyzing ubiquitin transfer rather than facilitating protein–protein interactions.

### The ATRN1–MGRN1 and ATRNL1–MGRN1 complexes ubiquitylate MC1R and MC4R

**Fig. 3.**
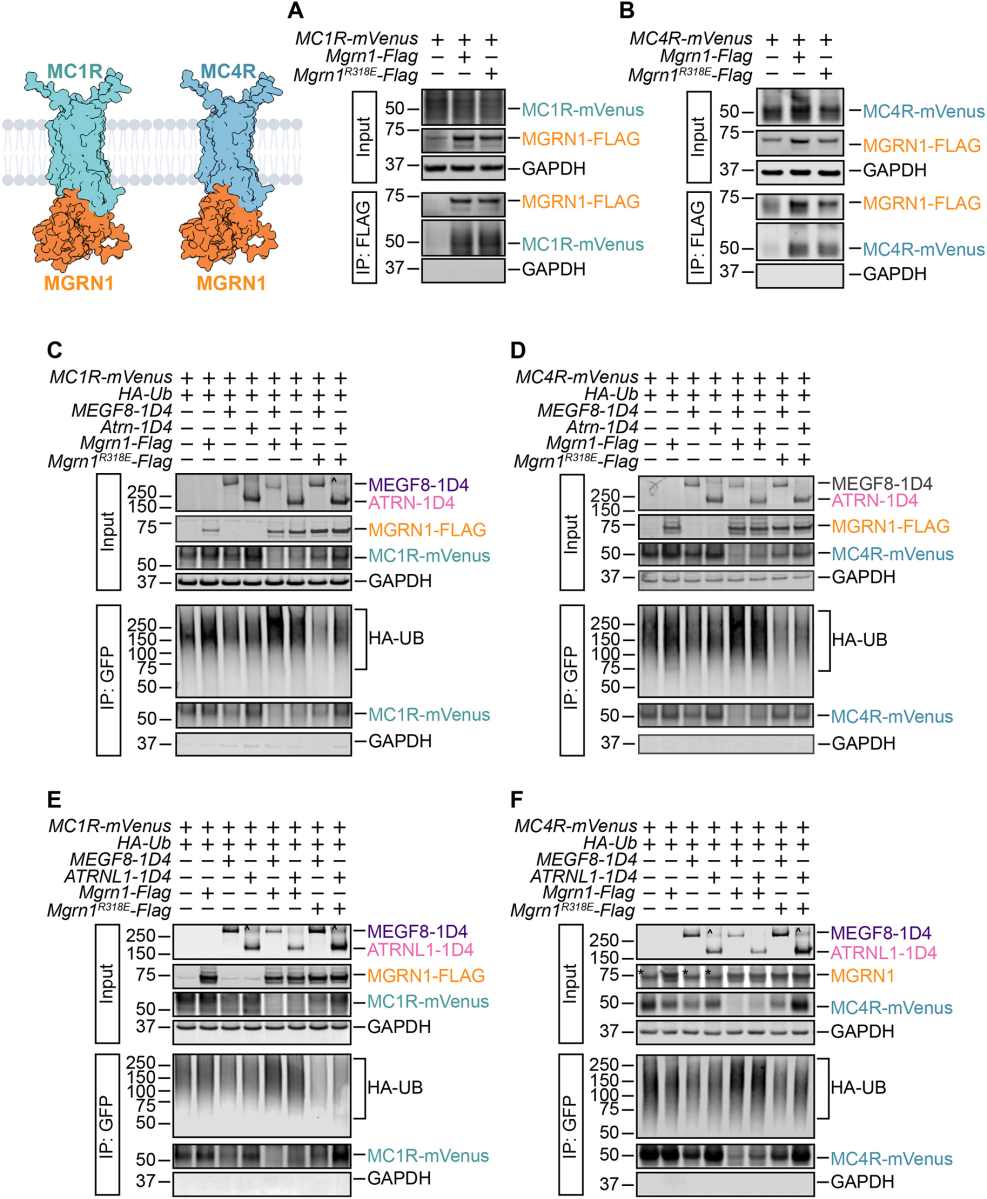
**MGRN1 interacts with MC1R and MC4R and facilitates their ubiquitylation.** (A, Left) AlphaFold-predicted MC1R–MGRN1 and MC4R–MGRN1 interactions. Protein structural models were computed using the AlphaFold2 Google Colab (Mirdita et al., 2022) using MC1R (UniProt ID: Q01726), MC4R (UniProt ID: P32245) and MGRN1 (UniProt ID: O60291) as inputs. (A,B) HEK293T cells were transfected with (A) *MC1R-Venus* or (B) *MC4R-Venus* along with either wild-type *Mgrn1-Flag* or the catalytically compromised *Mgrn1^R318E^-Flag*. We performed co-IP assays using anti-FLAG beads to pull down MGRN1 and then probed for either MC1R or MC4R using an anti-GFP antibody. In the absence of MGRN1–FLAG, MC1R and MC4R were not detected in the pulldown. However, both wild-type MGRN1 and MGRN1^R318E^ efficiently co-immunoprecipitated with MC1R and MC4R. (C–F) MC1R and MC4R ubiquitylation were assessed after transient coexpression of the indicated proteins in HEK293T cells. Briefly, HEK293T cells were co-transfected with (C,E) *MC1R-Venus* or (D,F) *MC4R-Venus* along with HA-tagged ubiquitin (*HA-Ub*), FLAG-tagged *Mgrn1* (wild-type or the *Mgrn1^R318E^* linchpin mutant), and the transmembrane adapter *MEGF8-1D4*, *Atrn-1D4* or *ATRNL1-1D4*. Cells were lysed under denaturing conditions, and MC1R or MC4R was purified by IP using GFP-Trap beads. The amount of HA-Ub covalently conjugated to MC1R and MC4R was assessed using immunoblotting with an anti-HA antibody. The transmembrane adapter was probed for using an anti-1D4 antibody. GAPDH serves as a loading control. The carets (^) indicate a likely ATRN and ATRNL1 dimer or oligomer, as observed in previous studies (Walker, 2010). The asterisks (*) indicate the endogenous MGRN1 present in HEK293T cells when probed with an anti-MGRN1 antibody (F). Blots representative of three independent repeats.

We previously found that the MEGF8–MGRN1 complex attenuates Hedgehog signaling strength by ubiquitylating the GPCR SMO and facilitating its internalization and degradation (Kong et al., 2020). Given that MGRN1 interacts with MEGF8 in a manner similar to ATRN and ATRNL1 (Fig. 2), we speculated that the resulting ATRN–MGRN1 and ATRNL1–MGRN1 complexes might target other surface receptors. Epistasis experiments in mice have consistently shown that MGRN1 and ATRN inhibit melanocortin signaling through MC1R and MC4R (Miller et al., 1997; Overton and Leibel, 2011; Phan et al., 2002), as evidenced by changes in coat color and satiety. Thus, we focused on these two receptors. As previously reported (Pérez-Oliva et al., 2009), we first utilized co-IP assays in HEK293T cells to confirm that MGRN1 interacts with MC1R and MC4R (Fig. 3A,B). We then established an MCR ubiquitylation assay by expressing hemagglutinin (HA)-tagged ubiquitin (HA–Ub) and mVenus-tagged MC1R or MC4R in HEK293T cells, then measuring the amount of HA–Ub conjugated to the MCRs under denaturing conditions. The MCRs were isolated by IP, and the attached Ub chains were detected by immunoblotting with an anti-HA antibody. Co-expression of MGRN1 alone revealed no increase in MCR ubiquitylation over baseline. Similarly, co-expression of MEGF8 or ATRN or ATRNL1 alone showed no rise in MCR ubiquitylation over baseline. However, the co-expression of both MEGF8 and MGRN1, ATRN and MGRN1, or ATRNL1 and MGRN1 increased MCR ubiquitylation and concomitantly reduced MCR abundance (Fig. 3C–F). To validate these findings, we also included the catalytically compromised MGRN1^R318E^ construct, which failed to promote MCR ubiquitylation and degradation (Fig. 3C–F). Collectively, these results suggest that MGRN1 uses a transmembrane surface adapter (MEGF8, ATRN or ATRNL1) to target the MCRs. Importantly, our data also shows that MCR degradation is dependent on the ubiquitin ligase activity of MGRN1.

### Loss of *Mgrn1* promotes the accumulation of MC4R at the cell surface and primary cilium

**Fig. 4.**
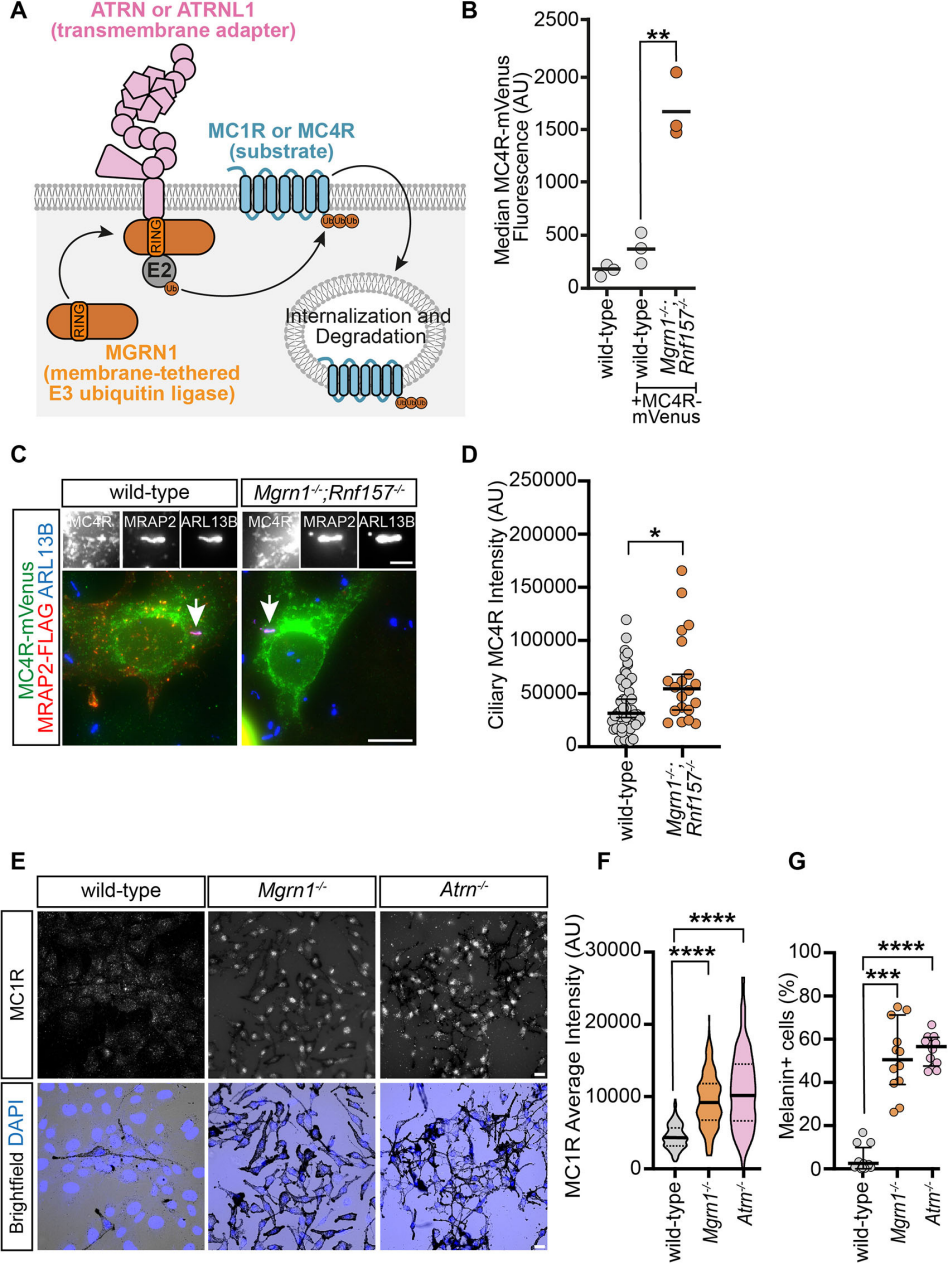
**Regulation of melanocortin receptors by MGRN1.** (A) A model depicting MGRN1 binding to the cytoplasmic tail of the transmembrane adapters ATRN or ATRNL1 to ubiquitylate the substrates MC1R or MC4R. (B) Flow analysis of wild-type and *Mgrn1^−/−^*; *Rnf157^−/−^* NIH/3T3 cells with stably integrated *MC4R-mVenus*. Each dot represents one experimental replicate consisting of 10,000 cells. Data is represented as a scatter dot plot, with the line highlighting the median. **P<0.01 (unpaired two-tailed *t*-test). (C) Representative widefield microscopy images of wild-type and *Mgrn1^−/−^*; *Rnf157^−/−^* NIH/3T3 cells transiently transfected with *MC4R-mVenus* and *MRAP2-Flag*. ARL13B is used to identify primary cilia (arrows) and DAPI to identify the nuclei. Scale bars: 10 µm (main images); 2 µm (upper panels). (D) Quantification of ciliary MC4R–mVenus in transfected wild-type and *Mgrn1^−/−^*; *Rnf157^−/−^* NIH/3T3 cells, where the primary cilia are defined by ARL13B. Each point represents a single cilium. Approximately 20–50 cilia were analyzed per group. The line highlights the median. Error bars show a 95% c.i. *P<0.05 (Mann–Whitney test). (E) Representative widefield immunofluorescence and brightfield microscopy images of primary melanocytes collected from wild-type, *Mgrn1^−/−^* and *Atrn*^−/−^ mice. Scale bars: 10 µm. (F) Quantification of MC1R intensity in wild-type, *Mgrn1^−/−^* and *Atrn^−/−^* melanocytes. The violin plot represents ∼150–200 melanocytes analyzed in each group. Data is represented as a truncated violin plot. The bold horizontal line represents the median, with the adjacent dotted lines representing the first and third quartiles. ****P<0.0001 (Kruskal–Wallis test). (G) Quantification of the percentage of melanin+ melanocytes observed in wild-type, *Mgrn1^−/−^* and *Atrn^−/−^* samples. Each point represents a single sample containing ∼50–200 melanocytes. Data is represented as a scatter dot plot, with the line highlighting the median and error bars representing a 95% c.i. ***P<0.001; ****P<0.0001 (Kruskal–Wallis test). AU, arbitrary units.

If MGRN1 regulates the ubiquitylation and degradation of MC1R and MC4R (Fig. 3), its loss should result in increased surface localization of these receptors (Fig. 4A). To test this, we used NIH/3T3 cells. NIH/3T3 cells do not endogenously express *MC4R* (Kinnebrew et al., 2019). Thus, we first used a lentiviral system to express *MC4R-mVenus* in either wild-type or *Mgrn1^−/−^*; *Rnf157^−/−^* NIH/3T3 cells. Flow cytometry revealed that *Mgrn1^−/−^*; *Rnf157^−/−^* cells exhibited approximately three times higher MC4R-mVenus fluorescence than wild-type cells, suggesting that a loss of these E3 ligases results in increased abundance of MC4R (Fig. 4B). To further assess this, we transiently co-transfected *MC4R-mVenus* and *MRAP2-Flag* into wild-type and *Mgrn1^−/−^*; *Rnf157^−/−^* NIH/3T3 cells. Melanocortin receptor-associated protein 2 (MRAP2) has been shown to enhance MC4R ciliary localization in both neurons and inner medullary collecting duct 3 (IMCD3) cells (Bernard et al., 2023). Consistent with these studies, MRAP2 promoted MC4R ciliary localization in wild-type NIH/3T3 cells, where primary cilia were visualized using ARL13B staining (Fig. 4C). Notably, *Mgrn1^−/−^*; *Rnf157^−/−^* cells exhibited an increase in MC4R ciliary localization compared to controls (Fig. 4D). This increase in ciliary MC4R was accompanied by a similar increase in ciliary SMO (Fig. S2B). Collectively, these findings suggest that MGRN1 regulates the surface abundance of multiple ciliary localized GPCRs, including MC4R and SMO.

### Loss of *Mgrn1* or *Atrn* promotes the accumulation of MC1R in melanocytes

To investigate the role of MGRN1 in regulating MC1R, we analyzed primary melanocytes derived from wild-type, *Mgrn1^−/−^* and *Atrn^−/−^* mice (Bennett et al., 1987; Gunn et al., 2001; Hida et al., 2009). Melanocytes are specialized epidermal cells responsible for pigment production, a process that is modulated by MC1R signaling. Activation of cell surface MC1R initiates a signaling cascade that drives the production of eumelanin (black pigment), whereas the absence of MC1R signaling results in the production of pheomelanin (yellow pigment). The dark eumelanin pigmentation phenotype of *Mgrn1*- and *Atrn*-null mice is consistent with increased MC1R signaling. To assess levels of cell surface-bound MC1R, we performed immunofluorescence staining using an antibody directed against an extracellular epitope of MC1R under detergent-free conditions. This analysis revealed a significant increase in MC1R abundance in *Mgrn1^−/−^* and *Atrn^−/−^* melanocytes compared to that in wild-type controls (Fig. 4E,F). The loss of *Mgrn1* or *Atrn* also led to a dramatic increase in eumelanin production, as evidenced by the higher number of melanin-positive cells in *Mgrn1^−/−^* and *Atrn^−/−^* melanocytes (Fig. 4E,G). Collectively, these findings suggest that MGRN1 and ATRN play a crucial role in modulating the abundance of MC1R, thereby influencing pigmentation outcomes.

## DISCUSSION

Previous studies have suggested that MGRN1 inhibits MC1R and MC4R signaling independent of ubiquitylation by competing with Gαs proteins for receptor binding (Pérez-Oliva et al., 2009), but this model does not fully account for all the *in vivo* phenotypes observed in *Mgrn1* and *Atrn* mutant melanocytes and mice, as previously reviewed (Walker and Gunn, 2010). In contrast, our findings support an adapter-mediated model of substrate specificity in which MGRN1 interacts with ATRN or ATRNL1 to regulate melanocortin receptor abundance and signaling activity. This mechanism is analogous to how MGRN1 interacts with MEGF8 to regulate SMO abundance (Kong et al., 2020, 2021) and thus builds upon our understanding of the fundamental principles of membrane-tethered E3 ligases (Williams et al., 2025 preprint), illustrating that interactions with transmembrane adapters allow this unique group of E3s to selectively target surface receptors for ubiquitylation and degradation.

A major unresolved question is how these membrane-tethered E3 complexes are regulated. In the Wnt signaling pathway, R-spondin ligands bind to the extracellular domain of the transmembrane E3 ligases ZNRF3 and RNF43 to regulate the abundance of surface receptors (Hao et al., 2012; Koo et al., 2012). We previously speculated that the MMM complex might be regulated in a similar manner through ligands interacting with the extracellular domain of MEGF8, but to date, no such ligand has been identified. In contrast, ATRN is already known to interact with specific ligands, providing an opportunity to investigate whether these interactions influence ATRN–MGRN1-mediated degradation of melanocortin receptors. Agouti-signaling protein (ASIP) is a unique ligand because it binds to both ATRN and the melanocortin receptors MC1R and MC4R (He et al., 2001; Ollmann et al., 1998; Yang et al., 1997). Classically, ASIP is believed to function as a competitive antagonist, directly binding to MC1R and MC4R to inhibit receptor activity while also preventing receptor activation by agonists. The prevailing model is that ATRN acts as a co-receptor, facilitating the interaction of ASIP with MC1R and MC4R to inhibit signaling (He et al., 2001). However, emerging evidence suggests that ASIP might play a more complex regulatory role. Although purified ASIP binds to MC1R and MC4R with high affinity in biochemical assays (Blanchard et al., 1995; Willard et al., 1995), genetic studies have shown that loss of ATRN or MGRN1 disrupts ASIP-dependent MC1R and MC4R degradation, leading to the accumulation of these receptors at the cell surface (Overton and Leibel, 2011; Phan et al., 2006). These findings suggest that ASIP might act as an initiation factor for the ATRN–MGRN1 complex, either activating it directly or positioning it in proximity to target MC1R and MC4R for ubiquitylation and degradation. Future investigation into this model will help us understand the ligand-mediated regulation of membrane-tethered E3 ligases. Ultimately, understanding the role of ASIP in this process will not only clarify how membrane-tethered E3s are regulated but also provide a framework for designing synthetic protein binders that modulate signaling activity by controlling receptor abundance.

An additional unresolved question is how transmembrane adapters confer substrate specificity to MGRN1. In our ubiquitylation assays using overexpressed proteins, the MEGF8–MGRN1, ATRN1–MGRN1 and ATRNL1–MGRN1 complexes were all capable of targeting MC1R and MC4R. However, we suspect this interchangeability is a product of overexpression and does not reflect the biological specificity observed in tissues. The distinct, non-overlapping phenotypes of *Megf8* and *Atrn* mutant mice strongly suggest that MEGF8 and ATRN have unique *in vivo* functions. This is supported by our finding that *Atrn^−/−^* and *Atrnl1^−/−^* cells have no elevation in ciliary SMO, unlike *Megf8^−/−^* and *Mgrn1^−/−^*; *Rnf157^−/−^* NIH/3T3 cells (Kong et al., 2020; Pusapati et al., 2018) (Fig. S2B). One explanation for this discrepancy is that differences in transmembrane adapter expression levels or tissue distribution dictate substrate specificity. We see evidence of this with ATRN and ATRNL1. ATRNL1 is expressed at lower levels than ATRN, resulting in *Atrn*-null mice developing pigmentation defects, an elevated metabolic rate and neurodegeneration. However, when *Atrnl1* is overexpressed under a stronger promoter (β-actin), it can rescue *Atrn* loss, revealing that functional redundancy can be masked by differential gene dosage (Walker et al., 2007). Another possibility is that interaction with transmembrane adapters might change the localization of MGRN1. In this example, MEGF8 and ATRN could localize MGRN1 to distinct subcellular compartments, thus enabling selective access to specific substrates. Finally, it is also possible that interactions with transmembrane adapters might serve to activate MGRN1, ensuring that its E3 ligase activity is engaged only when appropriately localized. Such activation could involve conformational changes or post-translational modifications triggered by the adapter, acting as a safeguard to prevent off-target ubiquitylation. In summary, overexpression might mask a more nuanced selection preference, highlighting the need for further exploration in endogenous systems.

Collectively, our findings provide a framework for understanding how MGRN1, as part of a membrane-tethered complex, regulates surface receptor abundance. This work builds on the concept of transmembrane adapter-mediated ubiquitylation, offering a new perspective on how MGRN1 functions within signaling pathways. Further investigation into the role of transmembrane adapters will be crucial for deciphering the molecular mechanisms underlying the diverse functions of MGRN1 and for exploring its potential as a target to regulate aberrant signaling.

## MATERIALS AND METHODS

### Reagents and antibodies

The following primary antibodies were used: mouse monoclonal anti-1D4 [The University of British Columbia, RRID: AB_325050, western blotting (WB) dilution 1:2000], guinea pig polyclonal anti-ARL13B (Dorn et al., 2012) [immunofluorescence (IF) dilution 1:1000], mouse monoclonal anti-ARL13B [Aves Labs, Cat #75287, RRID: AB_2341543, IF dilution 1:1000], rabbit polyclonal anti-ATRN (generated against a peptide from the cytoplasmic tail of ATRN, from Gregory Barsh, Department of Genetics and Pediatrics, Stanford University, CA, USA, WB dilution 1:1000; Walker, 2010), sheep polyclonal anti-ATRN (R&D systems, Cat #AF7238, RRID: AB_2843678, WB dilution 1:1000), rabbit polyclonal anti-ATRNL1 (Thermo Fisher Scientific, Invitrogen, Cat #PA5-90091, RRID: AB_2805919, WB dilution 1:1000), chicken polyclonal anti-FLAG (Aves Labs, Cat #ET-DY100, RRID: 2313510, IF and WB dilutions 1:1000), chicken polyclonal anti-GFP (Aves Labs, Cat #GFP-1010, RRID: AB_2307313, IF dilution 1:1000, WB dilution 1:2000), rabbit anti-GAPDH (LI-COR, Cat #926-42216, RRID: AB_2814901, WB dilution 1:10,000), rabbit polyclonal anti-HA (Proteintech, Cat #51064-2-AP, RRID: AB_11042321, WB dilution 1:2000), mouse monoclonal anti-HA (Proteintech, Cat #66006-2-Ig, RRID: AB_2881490, WB dilution 1:2000), mouse monoclonal anti-HA (Thermo Fisher Scientific, Invitrogen, Cat #26183, RRID: AB_10978021, WB dilution 1:2000), rabbit polyclonal anti-MEGF8 (WB dilution 1:2000; Kong et al., 2020), rabbit polyclonal anti-MC1R (Proteintech, Cat #26471-1-AP, RRID: AB_2880529, IF dilution 1:1000), rabbit polyclonal anti-MC1R (Millipore Sigma, Cat #AB5126, RRID: AB_91692, IF dilution 1:1000), rabbit polyclonal anti-RNF156 (anti-MGRN1, Proteintech, Cat #11285-1-AP, RRID: AB_2143351, WB dilution 1:1000) and rabbit polyclonal anti-SMO (IF dilution 1:1000; Rohatgi et al., 2007). Secondary antibodies conjugated to IRDye^®^ infrared dyes for western blotting or Alexa Fluor dyes for immunofluorescence were purchased from LI-COR, Thermo Fisher Scientific and Jackson Laboratories. We validated the ATRN and ATRNL1 antibodies by transfecting HEK293T cells with plasmids expressing their respective proteins and confirmed that each antibody is able to specifically recognize its intended target (Fig. S3).

### Constructs

*MEGF8-1D4*, *Mgrn1-Flag, Mgrn1^R318E^-Flag, Mosmo-HA* (featured in Figs 1D and 3A–F) were all cloned into the pEF5/FRT/V5-DEST expression vector using Gateway recombination methods (Thermo Fisher Scientific, Invitrogen) and are as previously described in earlier papers (Kong et al., 2020; Pusapati et al., 2018). *pRK5-HA-Ubiquitin-WT* (referred to as ‘HA–Ub’ in Fig. 3C–F) was Addgene #17608 (Lim et al., 2005). Full-length mouse *Mgrn1* (NM_001252437.1) with a C-terminal 3×FLAG tag was synthesized as a gBlock (Integrated DNA Technologies) and used as a template for the generation of all *Mgrn1* constructs. Overlap extension PCR was used to generate *Mgrn1^R318E^*, *Mgrn1^ΔPreEngShort^* [deletion of amino acids (aa) 35–48], *Mgrn1^ΔPreEngLong^* (deletion of aa 35–82), *Mgrn1^ΔEng^* (deletion of aa 79–260), *Mgrn1^ΔRING^* (deletion of aa 260–335), and *Mgrn1^ΔTail^* (deletion of aa 355–460) (featured in Figs 1–3). *Atrn-1D4* was generated through the PCR amplification of full-length mouse *Atrn* from an *Atrn-GFP* construct (Walker, 2010) and tagged with a C-terminal 1D4 (featured in Figs 1D, 3C,D). Full-length human *ATRNL1* (NM_207303.4) was PCR amplified from *pENTR223.1_ATRNL1* (Horizon Discovery) and similarly tagged with a C-terminal 1D4 (featured in Figs 1D, 3E,F). All the *Mgrn1, Atrn* and *ATRNL1* constructs were initially cloned into the pENTR2B plasmid (Thermo Fisher Scientific, Invitrogen) and then cloned into pEF5/FRT/V5-DEST expression vector (Thermo Fisher Scientific, Invitrogen) using Gateway recombination methods (Thermo Fisher Scientific, Invitrogen). *MC1R-mVenus* and *MC4R-mVenus* were originally generated in the laboratory of Christian Siebold (University of Oxford, UK). Briefly, human *MC1R* (UniProt ID. Q01726) and *MC4R* (UniProt ID. P42127) genes were codon optimized and synthesized (GeneArt, Thermo Fisher Scientific). The N-terminal methionine was removed before cloning into the pHR-CMV-TetO2 vector (Elegheert et al., 2018) in frame to a C-terminal 3C protease cleavage site followed by mVenus (Nagai et al., 2002; Zacharias et al., 2002) and TwinStrep (Schmidt et al., 2013) tags. *MRAP2-Flag* was a gift from Maxence Nachury (University of California, San Francisco, USA). *MC1R-mVenus*, *MC4R-mVenus*, and *MRAP2-Flag* were all cloned from their respective parent plasmids into pENTR2B (Thermo Fisher Scientific, Invitrogen) and then into the pEF5/FRT/V5-DEST expression vector (Thermo Fisher Scientific, Invitrogen) using Gateway recombination methods (Thermo Fisher Scientific, Invitrogen) (featured in Figs 3 and 4).

### Cell culture

The HEK293T cell line was purchased from the American Type Culture Collection (ATCC #CRL-1573) and the cells were originally isolated from the kidney of a human female embryo. The HEK293FT cell line (Cat #R70007) and the Flp-In™-3T3 cell line (Cat #R76107) were both purchased from Thermo Fisher Scientific. The HEK293FT cell line is a derivative of HEK293T cells. The Flp-In™-3T3 cell line is a derivative of NIH/3T3 cells, a mouse fibroblast cell line isolated from a female mouse NIH/Swiss embryo (ATCC #CRL-1658), and throughout the text we refer to these as ‘NIH/3T3 cells’. The HEK293T and NIH/3T3 cells were cultured in complete medium, composed of Dulbecco's modified Eagle medium (DMEM) containing high glucose (Cytiva, Cat #SH30081.FS) and supplemented with 10% fetal bovine serum (FBS) (Thermo Fisher Scientific, Cat #A5256701), 1× GlutaMAX supplement (Gibco, Cat #35050061), 1 mM sodium pyruvate (Gibco, Cat #11360070), 1× MEM non-essential amino acids solution (Gibco, Cat #11140076) and 1× penicillin-streptomycin (Gibco, Cat #15140163). The NIH/3T3 and HEK293T cells were passaged with 0.05% Trypsin-EDTA with Phenol Red (Gibco, Cat #25300062).

Wild-type melan-a melanocytes were originally a gift from Dorothy Bennett (Department of Cell Biology, St George’s, University of London, London, UK). *Mgrn1^md-nc/md-nc^* (melan-md3) and *Atrn^mg-3J/mg-3J^* (melan-mg1) null mutant melanocytes were originally generated by Elena Sviderskaya at the Wellcome Trust Functional Genomics Cell Bank, in collaboration with Drs Gregory Barsh and Dorothy Bennett as described previously (Hida et al., 2009). Immortalized melanocytes were cultured as previously reported with minor modifications (Bennett et al., 1987). Briefly, the melanocytes were cultured on six-well polystyrene plates coated with 0.1% gelatin (Sigma-Aldrich, Cat #501785182) in RPMI 1640 basal medium (Gibco, Cat #11875093) supplemented with 1× GlutaMAX supplement (Gibco, Cat #35050061), 1× penicillin-streptomycin (Gibco, Cat #15140163), 10% FBS (Thermo Fisher Scientific, Cat #A5256701), and 200 nM 12-*O*-tetradecanoyl phorbol-13-acetate (TPA) (Millipore Sigma, Cat #5005820001). Melanocytes were passaged upon reaching 80–85% confluency using 0.05% Trypsin-EDTA with Phenol Red (Gibco, Cat #25300062). All cells were housed at 37°C in a humidified cell culture incubator containing 5% CO_2_. All cell lines and new lines derived from these cells were free of mycoplasma contamination as determined by PCR using the Universal Mycoplasma Detection Kit (ATCC, Cat #30-1012K).

Clonal knockout NIH/3T3 lines carrying deletions in *Atrn* and *Atrnl1* using a dual guide strategy as previously reported (Pusapati et al., 2018). Briefly, two sgRNAs targeting either *Atrn* (ENSMUSG00000027312) or *Atrnl1* (ENSMUSG00000054843) were designed using Benchling with an interval spanning 20-100 bases. These guides were cloned into pSpCas9(BB)-2A-GFP (PX458, Addgene #48138; Ran et al., 2013) and pSpCas9(BB)-2A-mCherry. NIH/3T3 cells were co-transfected with Lipofectamine 3000 (Thermo Fisher Scientific, Invitrogen). GFP and mCherry double-positive single cells were sorted into a 96-well plate. Clonal lines were then screened by PCR using primers outside the cut region. The truncated bands were gel-purified using the Zymoclean^TM^ Gel DNA Recovery Kit (Cat #D4007) and subsequently sequenced to identify nonsense mutations (Fig. S2A). Sequences of primers and gRNAs were: *Atrn* sgRNA #1: 5′-TGGGTTGGTCTTCGGAAGAT-3′; *Atrn* sgRNA #2: 5′-TTGTCAGAGCCTAGTACTCG-3′; *Atrn* amplicon sequencing primer Fwd: 5′-TGGGGAATAGATGGTTTCCACAT-3′; Atrn amplicon sequencing primer Rev: 5′-TCTCCCTGCTGCCATAAGTA-3′; *Atrnl1* sgRNA #1: 5′-GGTGCTGTGGAGGGCTCGGC-3′. *Atrnl1* sgRNA #2: 5′-CCTGGCTGCTGCTGGACGGG-3′; *Atrnl1* amplicon sequencing primer Fwd: 5′-CCGTTGAGGAGCCGAGGAAC-3′; and *Atrnl1* amplicon sequencing primer Rev: 5′-TCCCACGGTAGCACTTACTTGA-3′. The *Atrn* guides targeted exon 16, to induce a frameshift mutation and premature stop, akin to that observed in the *Atrn^mg-3J/mg-3J^* (mahogany) mouse mutant (Miller et al., 1997).

### Generation of stable NIH/3T3 cell lines expressing desired transgenes

Clonal *Mgrn1^-/−^; Rnf157^−/−^* NIH/3T3 cells were previously generated and validated (Kong et al., 2020). Flp recombinase-mediated DNA recombination (Thermo Fisher Scientific, Invitrogen) was used to stably express wild-type *Mgrn1-1D4*, *Mgrn1-Flag*, *Mgrn1^ΔPreEngShort^-Flag*, *Mgrn1^ΔPreEngLong^-Flag*, *Mgrn1^ΔEng^-Flag*, *Mgrn1^ΔRING^-Flag*, *Mgrn1^ΔTail^-Flag*, *Mgrn1^R318E^-Flag,* or *MC4R-mVenus* in wild-type and *Mgrn1^−/−^; Rnf157^−/−^* NIH/3T3 cells. Briefly, wild-type and *Mgrn1^−/−^; Rnf157^−/−^* NIH/3T3 cells were transfected with 2.7 µg pOG44 Flp-recombinase expression vector (Thermo Fisher Scientific, Invitrogen) and 0.3 µg of the gene of interest (cloned into the pEF5/FRT/V5-DEST expression vector) using the X-tremeGENE™ 9 DNA Transfection Reagent (Roche Molecular Systems, Cat #6365787001) diluted in Opti-MEM^TM^ Reduced Serum Medium (Gibco, Cat #31985062). After 48 h, the cells were passaged onto 10 cm plates and selected with complete medium supplemented with 200 µg/ml Hygromycin B antibiotic (Gibco, Cat #10687010). The medium containing the antibiotic was replenished every 3–4 days, and selection continued for ∼2 weeks or until all cells on the control non-transfected plate had died.

### Transient expression of desired constructs in NIH/3T3 cells

In a 24-well plate, 100,000 cells of either wild-type, *Mgrn1^−/−^; Rnf157^−/−^; Atrn^−/−^*, and *Atrnl1^−/−^* NIH/3T3 cells were plated on glass coverslips in 500 µl of antibiotic-free complete medium (complete medium without the addition of 1× penicillin-streptomycin). Approximately 1 h after plating, desired constructs (*MC4R-mVenus* and *MRAP2-Flag*) cloned into the pEF5/FRT/V5-DEST expression vector were transiently transfected into the cells using X-tremeGENE™ 9 DNA Transfection Reagent (Roche Molecular Systems, Cat #6365787001) diluted in Opti-MEM^TM^ reduced serum medium (Gibco, Cat #31985062). To facilitate ciliation, after the cells were confluent, the cells were transitioned to low-serum medium (complete medium containing 0.5% FBS) overnight.

### Mass spectrometry

Protein sequence analysis by liquid chromatography-mass spectrometry (LC-MS) was conducted at the Taplin Biological Mass Spectrometry Facility (Harvard Medical School). Briefly, excised gel bands were cut into ∼3 mm pieces. Gel pieces were then subjected to a modified in-gel trypsin digestion procedure (Shevchenko et al., 1996). Gel pieces were washed and dehydrated with acetonitrile for 10 min, and then the acetonitrile was removed. Gel pieces were then completely dried in a speed vac. The gel pieces were then rehydrated with a 50 mM ammonium bicarbonate solution containing 12.5 ng/µl modified sequencing-grade trypsin (Promega) at 4°C. After 45 min, the excess trypsin solution was removed and replaced with 50 mM ammonium bicarbonate solution at a sufficient volume to cover the gel pieces. Samples were then placed in a 37°C room overnight. Peptides were later extracted by removing the ammonium bicarbonate solution, followed by one wash with a solution containing 50% acetonitrile and 1% formic acid. The extracts were then dried in a speed vacuum for ∼1 h. The samples were then stored at 4°C until analysis. On the day of analysis, the samples were reconstituted in 5–10 µl of HPLC solvent A (2.5% acetonitrile, 0.1% formic acid). A nano-scale reverse-phase HPLC capillary column was created by packing 2.6 µm C18 spherical silica beads into a fused silica capillary (100 µm inner diameter and ∼30 cm length) with a flame-drawn tip (Peng and Gygi, 2001). After equilibrating the column, each sample was loaded via a Famos auto sampler (LC Packings) onto the column. A gradient was formed, and peptides were eluted with increasing concentrations of solvent B (97.5% acetonitrile, 0.1% formic acid). As peptides eluted, they were subjected to electrospray ionization and then entered into a Velos Orbitrap Elite ion-trap mass spectrometer (Thermo Fisher Scientific). Peptides were detected, isolated and fragmented to produce a tandem mass spectrum of specific fragment ions for each peptide. Peptide sequences (and hence protein identity) were determined by matching protein databases with the acquired fragmentation pattern with the software program Sequest (Thermo Fisher Scientific) (Eng et al., 1994). All databases include a reversed version of all the sequences and the data was filtered to between a 1% and 2% peptide false discovery rate.

Analysis of interacting proteins (featured in Fig. 1B) was conducted at the University of Washington. For this process, the mass spectrometry raw data files were searched using a standardized workflow implemented in Nextflow and available at https://github.com/mriffle/nf-teirex-dda (revision:3 9f01e6119d57b34e3442fc2905ea7c63874926d). Briefly, the raw data files were converted into mzML using msconvert (Chambers et al., 2012) (ProteoWizard release: 3.0.22335), searched using comet (Eng et al., 2013) (version 2023010), post-processed with percolator (Käll et al., 2007) (version 3.05), and uploaded to Limelight (Riffle et al., 2022) for analysis and visualization. The comet parameters file and FASTA file used to search the data are available upon request as part of the Limelight project (Riffle et al., 2025; https://limelight.yeastrc.org/limelight/d/pg/project/137), associated with their respective searches.

### Protein sequence analysis

FASTA sequences of *Homo sapiens* MEGF8 (UniProt ID: Q7Z7M0), *Homo sapiens* ATRN (UnitProt ID: O75882), and *Homo sapiens* ATRNL1 (UnitProt ID: Q5VV63) were obtained from UniProtKB. Multiple sequence alignments (MSAs) were generated using Clustal Omega with default settings, employing seeded guide trees and HMM profile-profile (Madeira et al., 2024). The generated alignment was visualized using Jalview 2 (Waterhouse et al., 2009) and color-coded to indicate sequence conservation. Secondary structure prediction of MEGF8 was performed using the JPred secondary structure prediction tool (Drozdetskiy et al., 2015) (Fig. 1C).

### Co-immunoprecipitation and western blotting

HEK293T and NIH/3T3 cells were rinsed in chilled 1× phosphate-buffered saline (PBS, Gold Biotechnology, Cat #P-271-50), scraped off the plate, and then the cells were pelleted by spinning them down at 300 ***g*** for 5 min. The cell pellets were then lysed in Immunoprecipitation Lysis Buffer containing 50 mM Tris-HCl pH 8.0 (Gold Biotechnology, Cat #T-095-100 and #T-400-500), 150 mM NaCl (Thermo Fisher Scientific, Cat #7647-14-5), 1% NP-40 (United States Biological, Cat #N3500), 1 mM DTT (Gold Biotechnology, Cat #DTT100), and 1× SIGMA*FAST*™ protease inhibitor cocktail (Millipore Sigma, Cat #S8830). Cells were lysed for 1 h on a shaker at 4°C, supernatants were clarified by centrifugation at 16,000 ***g*** for 30 min, and protein concentrations were quantified using the Pierce™ BCA Protein Assay Kit (Thermo Fisher Scientific, Cat #23225). 1D4-tagged MGRN1, MEGF8, and ATRNL1 (featured in Fig. 1) were captured by a 1D4 antibody (The University of British Columbia) covalently conjugated to Dynabeads™ Protein A (Thermo Fisher Scientific, Invitrogen Cat #10002D). FLAG-tagged MGRN1 (featured in Fig. 2) was captured using anti-FLAG^®^ M2 Magnetic Beads (Millipore Sigma, Cat #M8823). Beads were added to the protein lysates and incubated overnight at 4°C. The beads were then washed thoroughly: once with Wash Buffer A (50 mM Tris-HCl pH 8.0, 150 mM NaCl, 1% NP-40, and 1 mM DTT), once with Wash Buffer B (50 mM Tris-HCl pH 8.0, 500 mM NaCl, 0.1% NP-40, and 1 mM DTT), and finally once with Wash Buffer C (50 mM Tris-HCl pH 8.0, 0.1% NP-40, and 1 mM DTT). The proteins were then eluted by resuspending samples in 2× protein sample loading buffer (LI-COR, Cat #928-40004) supplemented with 100 mM DTT and incubated at 37°C for 30 min. All samples were then run on NuPAGE Bis-Tris precast gels (Thermo Fisher Scientific). The resolved proteins were transferred onto an Immobilon^®^-FL PVDF membrane (Millipore Sigma, Cat #IPFL00010) using a wet/tank blotting system (Bio-Rad Laboratories). Membranes were imaged on a LI-COR Odyssey CLx imaging system.

For quantification of interactions between MGRN1 and endogenous ATRN, the integrated intensity of the protein bands of interest (ATRN) was measured using ImageStudio version 6 software (LI-COR Biosciences). Signal intensities were then normalized by dividing the intensities of immunoprecipitated proteins (ATRN) by the intensities of the respective FLAG-tagged MGRN1 and MGRN1 truncation mutants. To compare interactions across conditions, the ratios of normalized immunoprecipitated proteins from the *Mgrn1^−/−^; Rnf157^−/−^* with wild-type *Mgrn1-Flag* addback samples were normalized to a signal intensity of 100. In each experiment, the corresponding ratios of normalized immunoprecipitated proteins in *Mgrn1^−/−^; Rnf157^−/−^* with *Flag*-tagged *Mgrn1* truncated samples were calculated relative to the functional *Mgrn1^−/−^; Rnf157^−/−^* with *Mgrn1-Flag* NIH/3T3 samples (Fig. 2C,D). Uncropped images of western blots from this paper are shown in Fig. S4.

### Ubiquitylation assay

4×10^6^ HEK293T cells were plated onto a 10 cm plate. At 24 h after plating, the cells were transfected using PEI Prime (polyethylenimine hydrochloride, 1 mg/ml, Cat #Prime-AQ100-100ML). 3 µg of each construct was transfected into the cells (at a DNA:PEI ratio of 1:3). An empty plasmid construct was used as a filler to ensure that each plate was transfected with the same amount of DNA. At 48 h post-transfection, the cells were pre-treated with 10 µM Bortezomib (a proteasome inhibitor, LC Laboratories, Cat #B-1408) and 100 µM Chloroquine (a lysosome and autophagy inhibitor, Cayman, Cat #14194) for 4 h to enrich for ubiquitylated proteins. Cells were washed twice with chilled 1× PBS (Gold Biotechnology, Cat #P-271-50) and lysed in ubiquitylation lysis buffer A consisting of 50 mM Tris-HCl pH 8.0 (Gold Biotechnology, Cat #T-095-100 and #T-400-500), 150 mM NaCl (Thermo Fisher Scientific, Cat #7647-14-5), 2% NP-40 (United States Biological, Cat #N3500), 0.25% sodium deoxycholate (Thermo Fisher Scientific, Cat #J62288), 0.1% SDS (Thermo Fisher Scientific, Cat #28312), 6 M urea (Thermo Fisher Scientific, Cat #A12360), 1 mM DTT (GoldBio, Cat #DTT50), 10 µM Bortezomib, 100 µM Chloroquine, 20 mM N-Ethylmaleimide (NEM, Thermo Fisher Scientific, Cat # 23030), and 1× SIGMA*FAST* protease inhibitor cocktail (Millipore Sigma, Cat #S8820). Clarified supernatants were diluted 10-fold with ubiquitylation lysis buffer B (ubiquitylation lysis buffer A prepared without urea) to adjust the urea concentration to 600 mM. For these assays, we assessed ubiquitylation on GFP-tagged proteins. Ubiquitylated GFP-tagged MC1R (in Fig. 3C,E) and MC4R (in Fig. 3D,F) were captured using a GFP Nanobody/VHH coupled to magnetic agarose beads (ChromoTek GFP-Trap^®^ Magnetic Agarose, Proteintech Cat #gtma-20) rotating overnight at 4°C. The beads were then washed once with ubiquitylation wash buffer A (ubiquitylation lysis buffer B+0.5% SDS), once with ubiquitylation wash buffer B (ubiquitylation wash buffer A+1 M NaCl), and finally once again with ubiquitylation wash buffer A. Proteins bound to the magnetic beads were eluted in 2× LDS sample buffer (LI-COR, Cat #928-40004) containing 30 mM DTT at 37°C for 30 min and assayed by immunoblotting with anti-chicken-GFP (Aves Labs, Cat #GFP-1010) for GFP-tagged MC1R and MC4R and anti-mouse-HA (Proteintech, Cat #66006-2-lg) for HA-tagged ubiquitin conjugated GFP-tagged MC1R and MC4R.

### Immunofluorescence staining of cells and image quantifications

NIH/3T3 cells were fixed in chilled 4% paraformaldehyde (PFA; MP Biomedicals, Cat #0215014601) diluted in 1× PBS (Gold Biotechnology, Cat #P-271-50) for 10 min on ice, followed by 3 rinses (5 min each) with 1× PBS. Cells were then incubated for 1 h in blocking buffer comprising 1% horse serum (Cytiva, Cat #SH3007403) and 0.1% Triton X-100 (Thermo Fisher Scientific, Cat #BP151-100) diluted in 1× PBS. The cells were then incubated in primary antibodies overnight at 4°C and secondary antibodies for 1 h at room temperature (all antibodies were diluted in blocking buffer). Coverslips were mounted on slides using ProLong™ diamond antifade mountant with DAPI (Thermo Fisher Scientific, Invitrogen, Cat #P36962). Fluorescence images were captured on a Nikon eclipse Ti2 microscope equipped with a 20× air and 60× oil immersion objective (NA 1.42). *Z*-stacks (∼4 µm thick stacks) were acquired with uniform acquisition settings (laser power, gain, offset, frame and image format) within a given experiment.

Primary melanocytes were fixed in chilled 4% PFA (MP Biomedicals, Cat #0215014601) diluted in 1× PBS (Gold Biotechnology, Cat #P-271-50) for 10 min on ice, followed by 3 rinses (5 min each) with 1× PBS. Cells were then incubated for 1 h in detergent-free blocking buffer comprising 1% horse serum (Cytiva, Cat #SH3007403) diluted in 1× PBS. The cells were then incubated in primary antibodies overnight at 4°C and secondary antibodies for 1 h at room temperature (all antibodies were diluted in Detergent-Free Blocking Buffer). Coverslips were mounted on slides using ProLong™ diamond antifade mountant with DAPI (Thermo Fisher Scientific, Invitrogen, Cat #P36962). Fluorescent images were captured on a Nikon eclipse Ti2 microscope equipped with a 60× oil immersion objective (NA 1.42). *Z*-stacks (∼4 µm thick stacks) were acquired with uniform acquisition settings (laser power, gain, offset, frame and image format) within a given experiment.

For the quantification of SMO at cilia, .nd2 Nikon stacked images were opened in Fiji (Schindelin et al., 2012) with maximum intensity *Z*-projection. Ciliary masks were constructed based on the signal from ARL13B and then applied to corresponding SMO images to measure the fluorescence intensity of SMO at cilia. For ciliary MC4R quantification, ciliary masks were manually created using Fiji in only the transfected MC4R-mVenus+ cells guided by ARL13B. The masks were then applied to the MC4R channel to measure the fluorescence intensity of MC4R at cilia. For MC1R quantification, cell masks were manually created using Fiji. The masks were then applied to the MC1R channel to measure the mean intensity. For each image, a background mean intensity was calculated from a region with no cells, and this was subtracted from the signal calculated from each cell. Lastly, melanin-positive (melanin+) cells were counted using the Countess 3 Automated Cell Counter (Thermo Fisher Scientific).

### Hedgehog signaling assays in NIH/3T3 cells

For all Hedgehog signaling assays, NIH/3T3 cells were first grown to confluence in complete medium (containing 10% FBS), then the cells were transitioned to low-serum medium (complete medium containing 0.5% FBS) for 24 h to encourage ciliation. Hedgehog signaling activity was measured using real-time quantitative reverse transcription PCR (qRT-PCR) (Fig. S1C). Briefly, RNA was extracted from the cells using a Direct-zol™ RNA miniprep kit with TRI Reagent^®^ (Zymo Research, Cat #R2053). RNA concentrations were measured on a NanoDrop™ spectrophotometer (Thermo Fisher Scientific) and equal amounts of RNA were used as a template for cDNA synthesis using a High-Capacity cDNA Reverse Transcription Kit (Thermo Fisher Scientific, Applied Biosystems, Cat #4368814). qRT-PCR for *mGli1* and *mGapdh* was performed on a QuantStudio 5 real-time PCR System (Thermo Fisher Scientific) with the following custom designed primers: *mGli1* (Fwd 5′-CCAAGCCAACTTTATGTCAGGG-3′ and Rev 5′-AGCCCGCTTCTTTGTTAATTTGA-3′) and *mGapdh* (Fwd 5′-AGTGGCAAAGTGGAGATT-3′ and Rev 5′-GTGGAGTCATACTGGAACA-3′). *Gli1* transcript levels were calculated relative to *Gapdh* and reported as a fold change across cell lines using the comparative C_T_ method (ΔΔC_T_ method).

### Fluorescence-activated cell sorting

Wild-type and *Mgrn1*^−/−^, *Rnf157^−/−^* NIH/3T3 cells stably expressing *MC4R-mVenus* were generated using the Flp-In system (as described above). The cells were cultured in complete medium until they were 80–90% confluent. At this point, the cells were detached using 0.05% trypsin-EDTA. The detached cells were filtered through a 70 µm filter to obtain a single-cell suspension. Approximately 10^6^ cells were collected and centrifuged at 200 ***g*** for 5 min. The cell pellet was resuspended in PBS to remove residual culture media, and this wash step was repeated once. For fixation, the cells were treated with 4% PFA in PBS for 10 min on ice. Following fixation, the cells were washed again in PBS to remove any residual PFA and resuspended in 1 ml PBS. The expression of MC4R-mVenus was assessed using the BD FACSymphony™ A3 flow cytometer and the acquired data was analyzed with BD FlowJo™ software. Data was collected from ∼10,000 events per replicate (Fig. 4B).

### Quantification and statistical analysis

All data analysis and graphs were generated using GraphPad Prism 10. Violin plots were created using the ‘Violin Plot (truncated)’ appearance function. In Prism 10, the frequency distribution curves of the violin plots are calculated using kernel density estimation. By using the ‘truncated’ violin plot function, the frequency distributions shown are confined within the minimum to maximum values of the data set. On each violin plot, the median (central bold line) and quartiles (adjacent dotted lines, representing the first and third quartiles) are labeled (Fig. 4F; Figs S1B and S2B). Dot plots were created in Prism 10 using the ‘Scatter dot plot’ appearance function (Figs 2D, 4B,D,G; Fig. S1C). Statistical significance was determined using Prism 10, with details and *P*-values specified in the individual figure legends. *P*-values were reported using the following key: not-significant (ns) *P*-value >0.05, *P-value <0.05, **P-value <0.01, ***P-value <0.001, and ****P-value <0.0001. Additional figure details regarding the *n*-value and statistical test applied are reported in the individual figure legends.

### Additional software and AI tool use

The manuscript was written with grammatical assistance from Grammarly. Citations were generated using Paperpile. After using these services, the authors reviewed and edited the content as needed and take full responsibility for the content of the publication.

## Supplementary Material

10.1242/joces.264084_sup1 Supplementary information
