# Supplementary material for: The E3 ubiquitin ligase MGRN1 targets melanocortin receptors MC1R and MC4R via interactions with transmembrane adapters
Source: J Cell Sci. 2025 Dec 9;138(23):jcs264084. doi: 10.1242/jcs.264084 (PMC12752501; doi:10.1242/jcs.264084)
Supplement: Supplementary information [file joces-138-264084-s1.pdf]

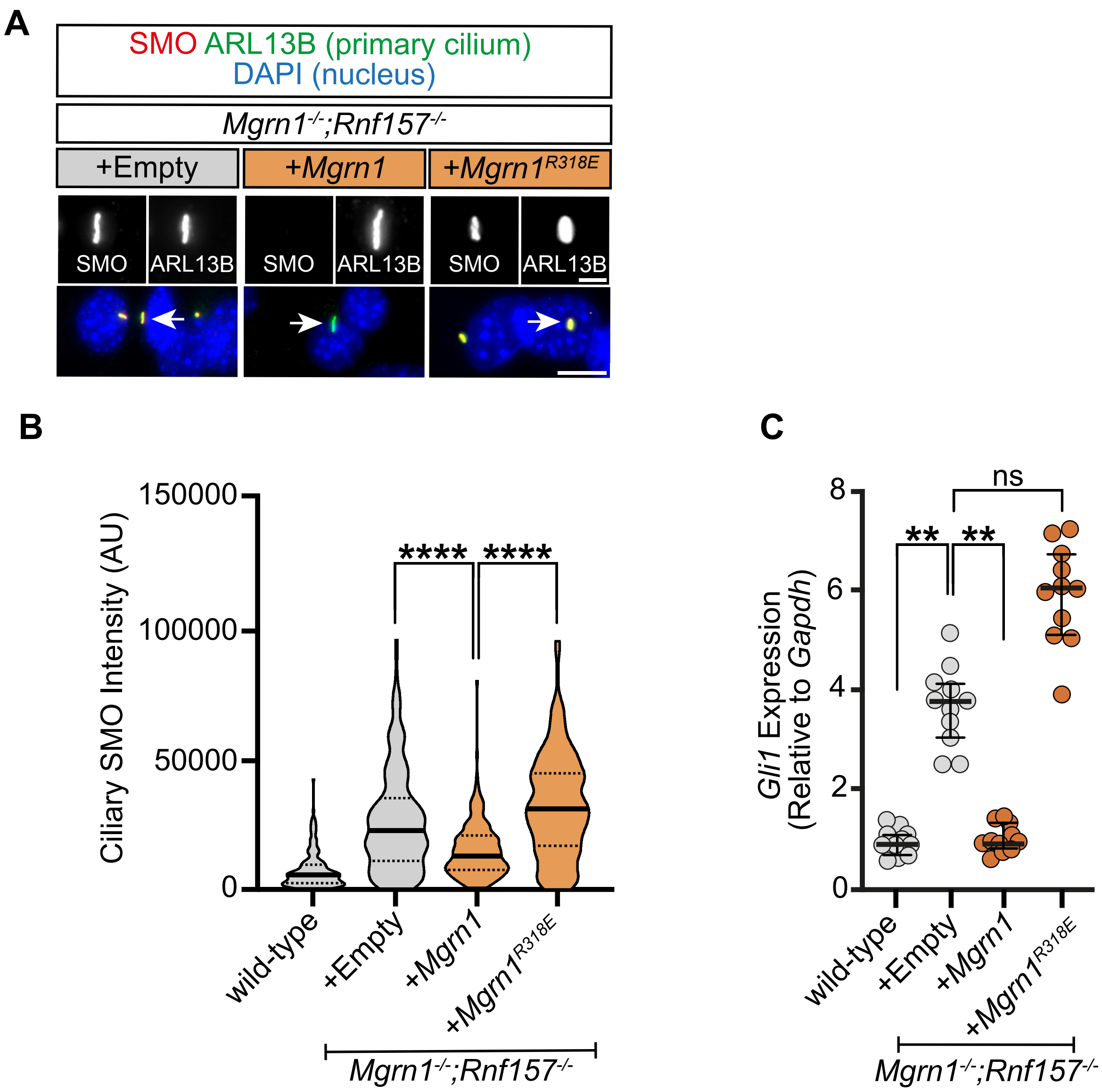

Fig. S1. Analysis of the linchpin mutant MGRN1^{R318E}

(A) Representative immunofluorescence widefield microscopy images of *Mgrn1*$^{-/-}$;*Rnf157*$^{-/-}$ NIH/3T3 cells stably expressing either Flag-tagged wild-type *Mgrn1* or catalytically compromised *Mgrn1*^{R318E}. SMO (red) is imaged at the primary cilia (green, marked by ARL13B), with DAPI (blue) used to label the nuclei. Arrows mark the primary cilium captured in the inset black-and-white images. Scale bars = 10 µm and 2µm (inset). **(B)** Hedgehog signaling strength was assessed by quantifying ciliary SMO in wild-type and *Mgrn1*$^{-/-}$;*Rnf157*$^{-/-}$ NIH/3T3 cells stably expressing either wild-type *Mgrn1* or catalytically inactive *Mgrn1*^{R318E}. Data is represented as a truncated violin plot of data collected from ~200 cilia analyzed in each group. Statistical significance was determined using the Kruskal-Wallis test. **** $p < 0.0001$. The bold horizontal line represents the median, with the adjacent dotted lines representing the first and third quartiles. **(C)** Hedgehog signaling strength was also assessed using qRT-PCR to measure *Gli1* mRNA, a direct Hedgehog target gene. The scatter dot plot represents mRNA values derived from 3-4 individual measurements collected from three experimental replicates. The bold horizontal line represents the median, with the adjacent thin lines representing the interquartile range. ** $p < 0.01$ and not-significant (ns).

Journal of Cell Science • Supplementary information

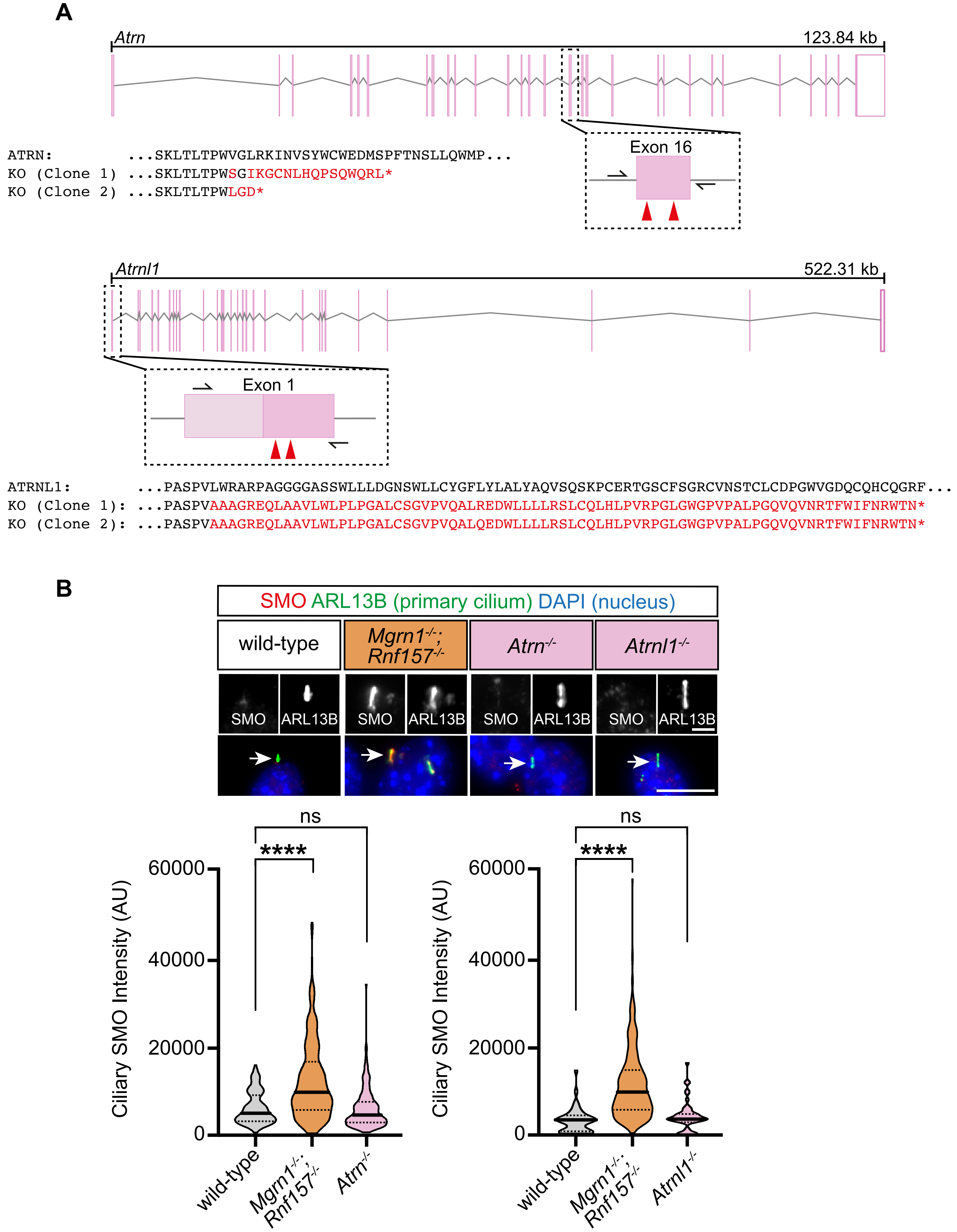

Journal of Cell Science • Supplementary information

Fig. S2. Generation and analysis of *Atrn*$^{-/-}$ and *Atrnl1*$^{-/-}$ NIH/3T3 cell lines

(A) Schematic diagrams of mouse *Atrn* and *Atrnl1* with insets highlighting the CRISPR/Cas9 targeted exons. Loss-of-function mutations were generated using a dual guide approach. In the exon insets, single-guide RNA (sgRNA) targets are highlighted in red and the location of peripheral primers used to screen for successful deletions are the black arrows. Included below each gene are the sequences from wild-type and knockout (KO) clones, showcasing the frameshift mutations (red text) that result in premature stop codons (*). **(B)** Representative immunofluorescence widefield microscopy images of primary cilia from wild-type, *Mgrn1*$^{-/-}$;*Rnf157*$^{-/-}$, *Atrn*$^{-/-}$, and *Atrnl1*$^{-/-}$ NIH/3T3 cells. SMO (red) is imaged at the primary cilia (green, marked by ARL13B), with DAPI (blue) used to label the nuclei. Arrows mark the primary cilium captured in the inset black-and-white images. Data is represented as a truncated violin plot of data collected from ~200 cilia analyzed in each group. Statistical significance was determined using the Kruskal-Wallis test. **** $p < 0.0001$. The bold horizontal line represents the median, with the adjacent dotted lines representing the first and third quartiles. Scale bars = 10 µm and 2µm (inset).

Journal of Cell Science • Supplementary information

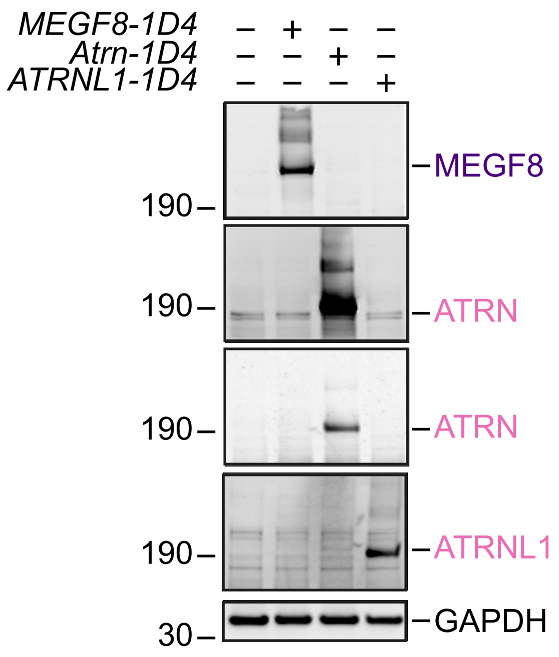

Fig. S3. Validation of ATRN and ATRNL1 antibodies

HEK293T cells were transfected with either an empty vector or 1D4-tagged *MEGF8*, *Atrn*, or *ATRNL1*. Whole-cell lysates were subjected to immunoblotting and probed (top to bottom) with anti-MEGF8 (rabbit polyclonal generated in the Rohatgi Lab), anti-ATRN (rabbit polyclonal generated in the Barsh Lab), anti-ATRN (sheep polyclonal purchased from R&D systems), and anti-ATRNL1 (rabbit polyclonal purchased from Invitrogen) antibodies. Each antibody recognized its corresponding protein without cross-reactivity, confirming the specificity of these antibodies towards their intended targets. GAPDH serves as a loading control.

Journal of Cell Science • Supplementary information

Figure 1D

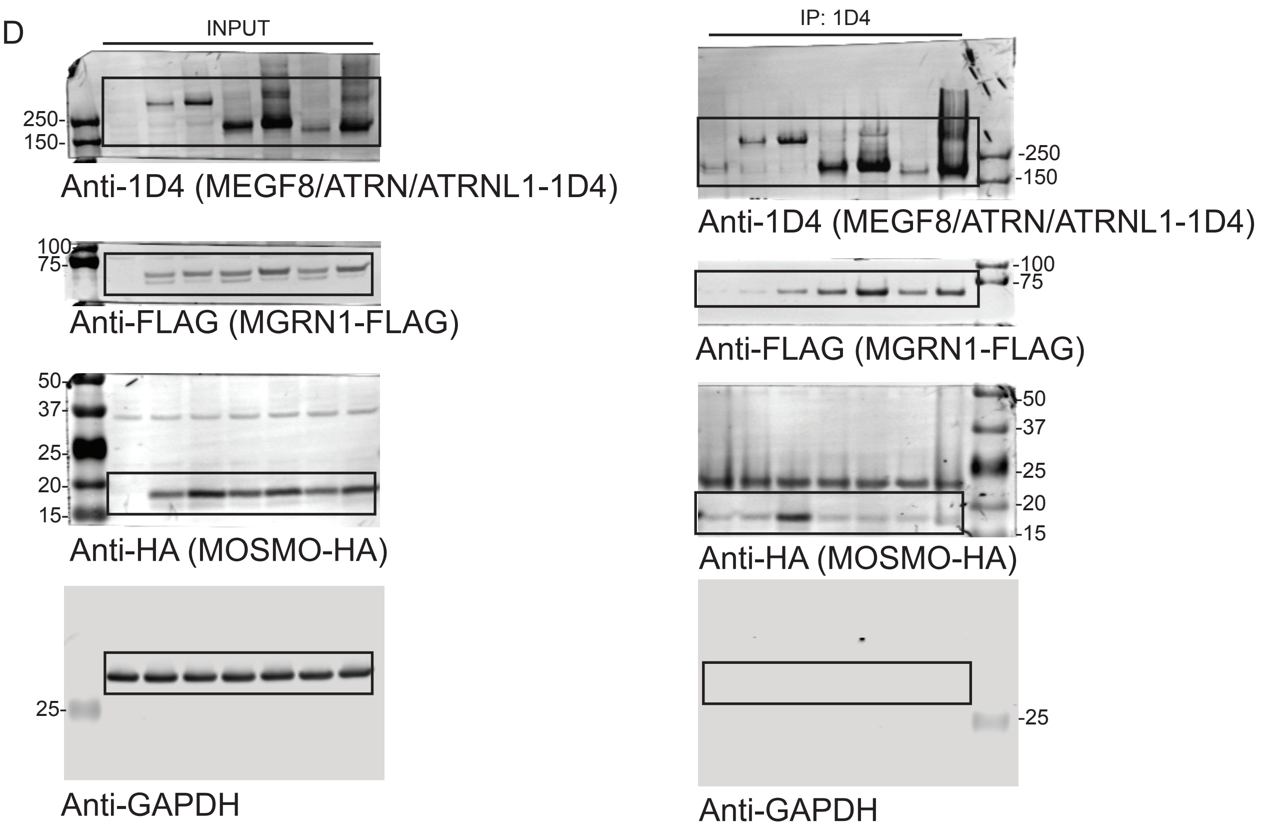

Figure 2C

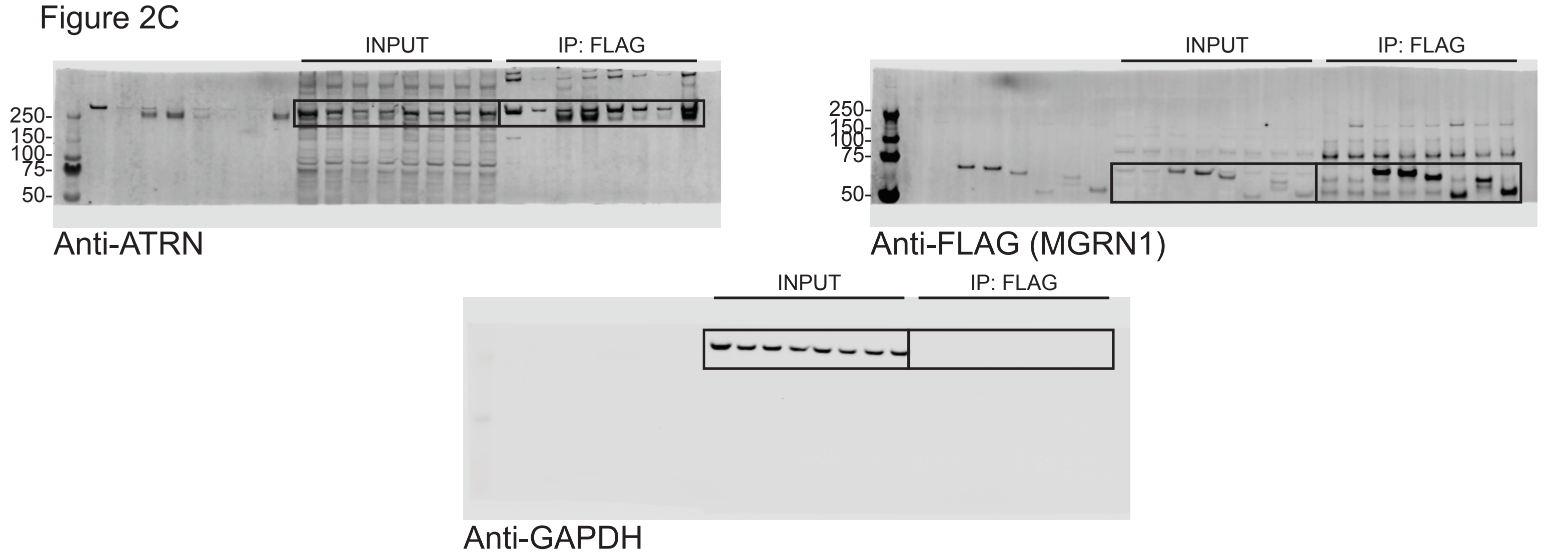

Figure 3A

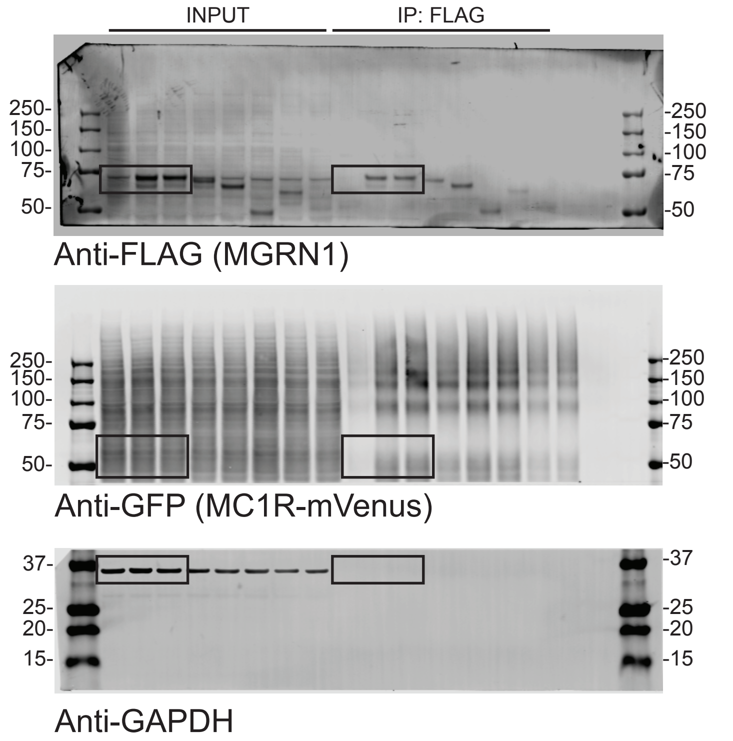

Figure 3B

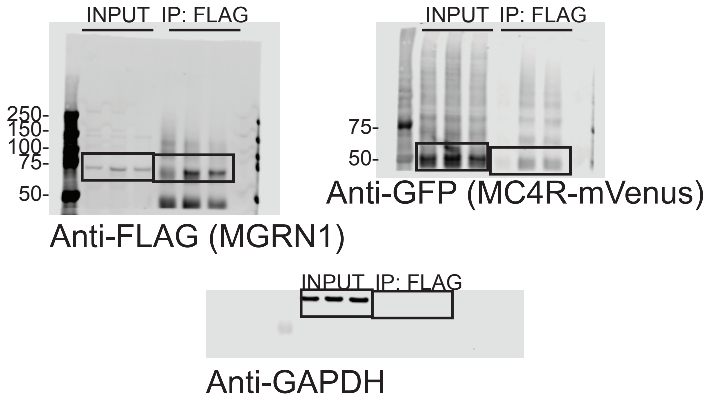

Journal of Cell Science • Supplementary information

Figure 3C

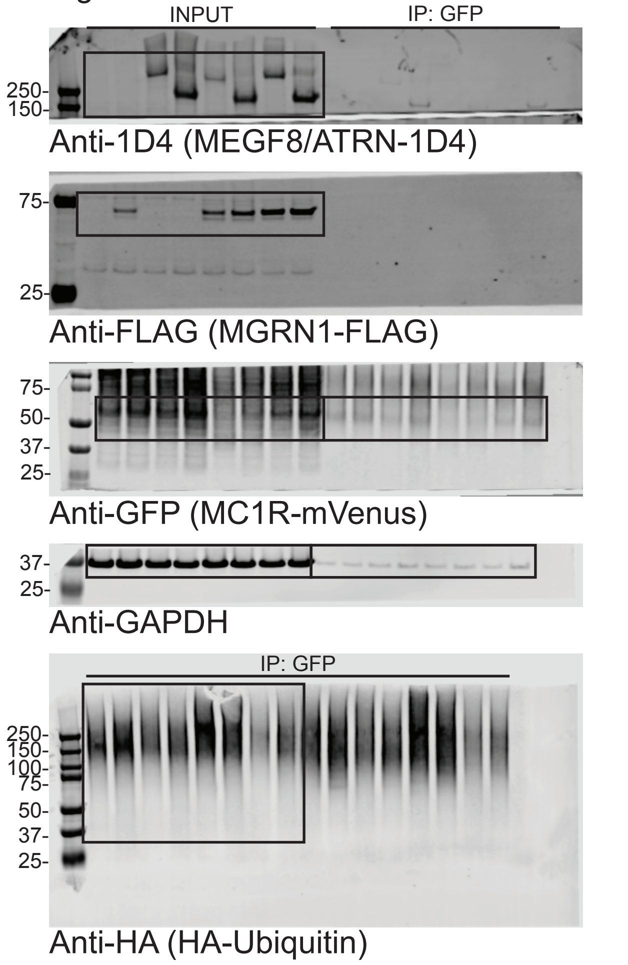

Figure 3D

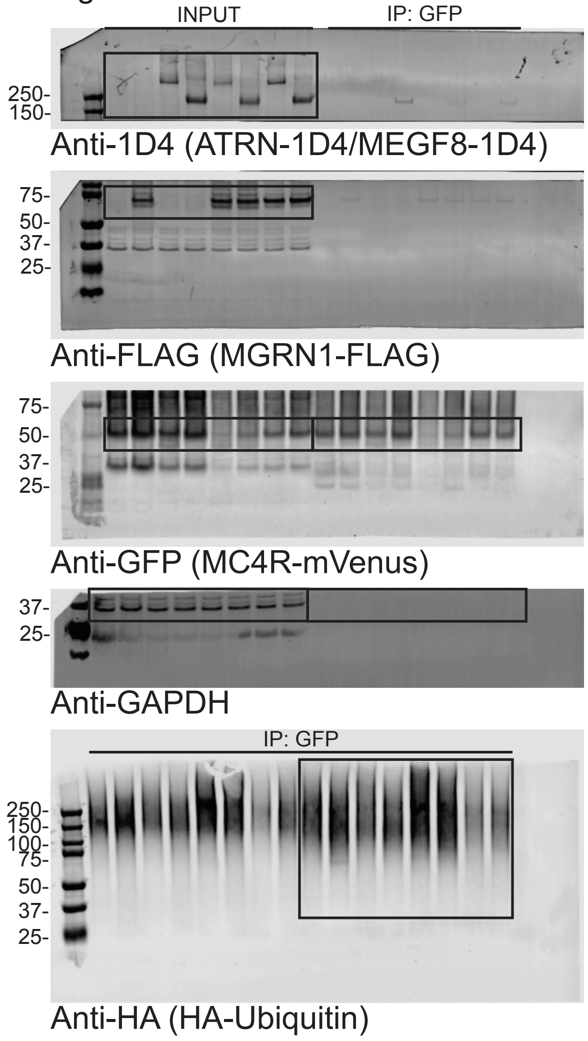

Figure 3E

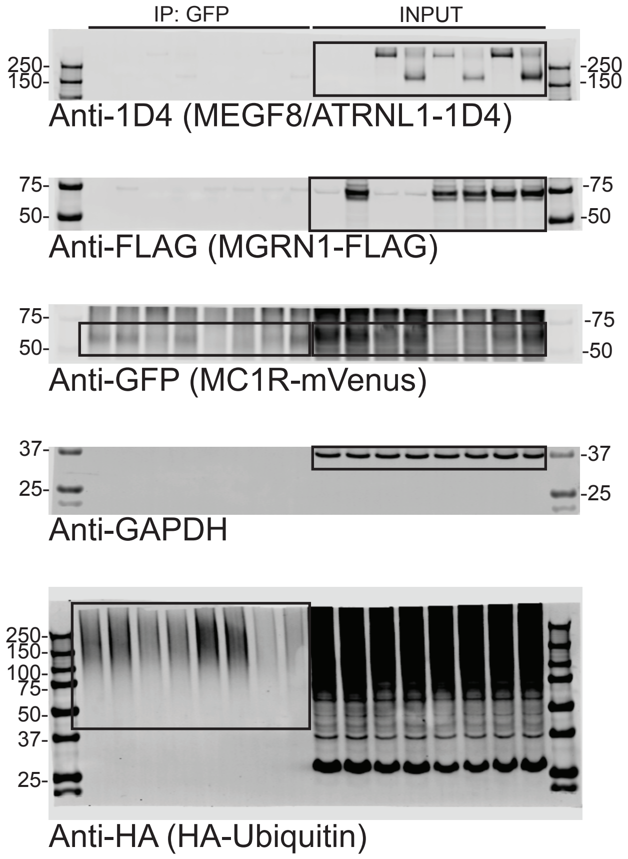

Figure 3F

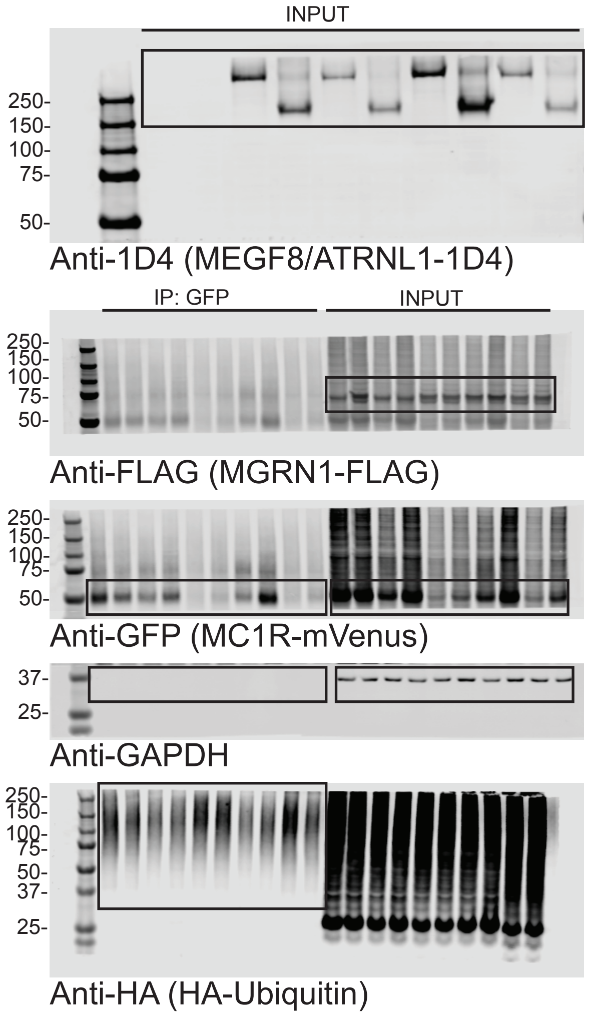

Journal of Cell Science • Supplementary information

Figure S3

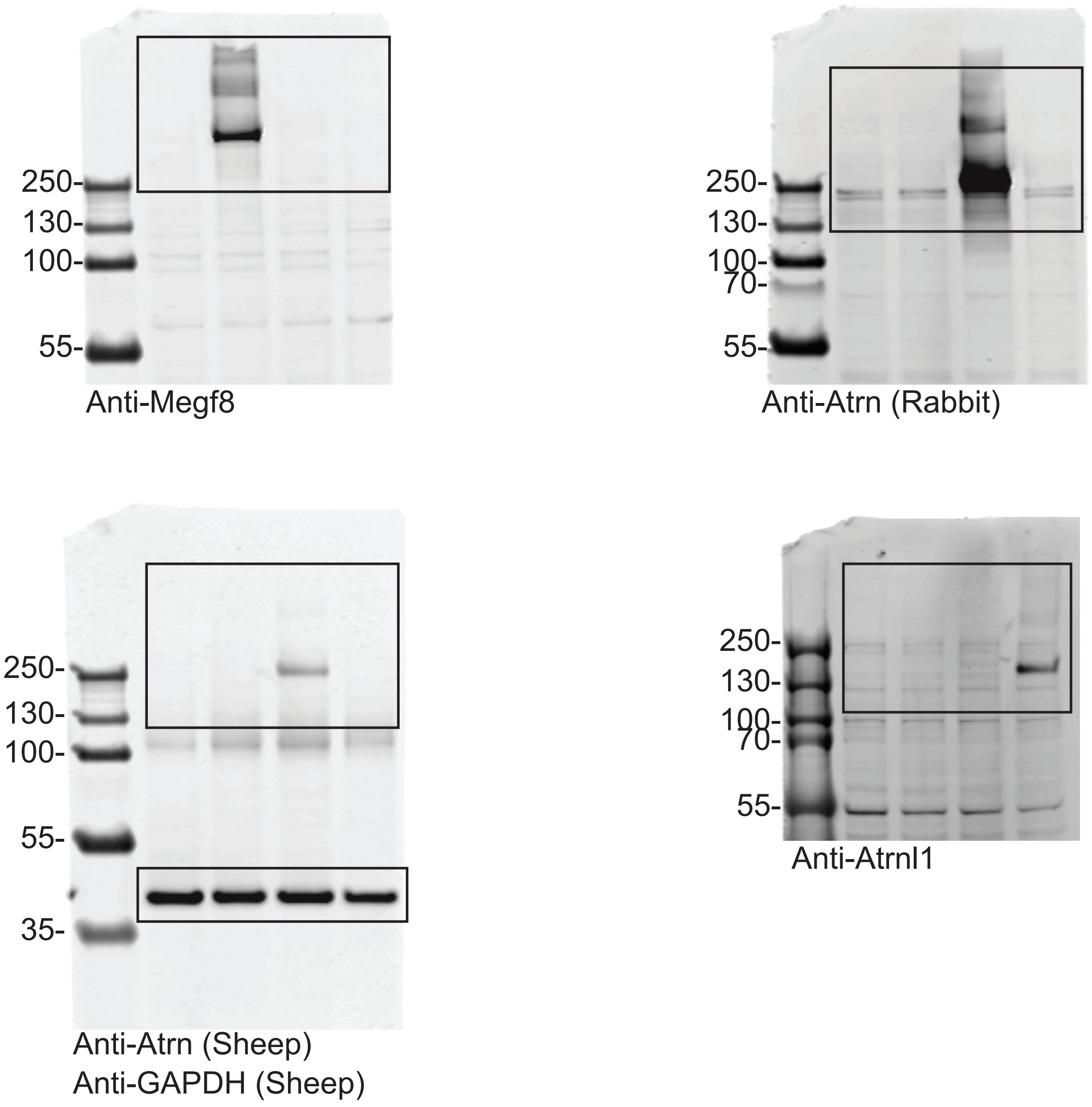

Fig. S4. Blot Transparency.

Journal of Cell Science • Supplementary information
